# Multivariate analysis of the summer herbaceous vegetation and environmental factors of the sub-tropical region

**DOI:** 10.1038/s41598-024-63780-8

**Published:** 2024-07-08

**Authors:** Tahseen Ullah, Zahir Muhammad, Ishaq Ali Shah, Mohammed Bourhia, Hiba-Allah Nafidi, Ahmad Mohammad Salamatullah, Youssouf Ali Younous

**Affiliations:** 1 Department of Botany, University of Peshawar, Peshawar, 25120 Pakistan; 2 Higher Education, Archives and Libraries Department, Government of Khyber Pakhtunkhwa, Peshawar, Pakistan; 3 Laboratory of Biotechnology and Natural Resources Valorization, Ibn Zohr University, Agadir, Morocco; 4 Department of Food Science, Faculty of Agriculture and Food Science, Laval University, Quebec City, QC Canada; 5 Department of Food Science and Nutrition, College of Food and Agricultural Sciences, King Saud University, Riyadh, Saudi Arabia; 6 Evangelical College, BP 1200, N’Djamena, Chad

**Keywords:** Multivariate analysis, Summer herbaceous vegetation, Kohat, Pakistan, Plant sciences, Ecology

## Abstract

Understanding the distribution of the plant species of an unexplored area is the utmost need of the present-day. In order to collect vegetation data, Quadrat method was used having size of 1 m^2^. The composite soil samples from each site were tested for various edaphic properties. PC-ORD v.5 was used for the classification of the vegetation while CANOCO v.5.1 was used for ordination of the data and to find out the complex relationship between plants and environment. Survey was conducted during summer season and a total of 216 herbaceous species were recorded from forty different sites of District Kohat, Pakistan. Cluster Analysis (CA) and Two-Way Cluster Analysis (TWCA) classified the vegetation of forty sites into six major plant groups i.e., 1. *Paspalum paspalodes, Alternanthera sessilis, Typha domingensis,* 2. *Cynodon dactylon, Parthenium hysterophorus, Brachiaria ramosa,* 3. *Cynodon dactylon, Eragrostis minor, Cymbopogon jwarancusa,* 4. *Cymbopogon jwarancusa, Aristida adscensionis*, *Boerhavia procumbens,* 5. *Cymbopogon jwarancusa, Aristida adscensionis, Pennisetum orientale* and 6. *Heteropogon contortus*, *Bothriochloa ischaemum*, *Chrysopogon serrulatus*. They were named after the dominant species based on their Importance Value (IV). The detrended correspondence analysis (DCA) analysis further confirmed the vegetation classification. Canonical correspondence analysis (CCA) indicated that the species distribution in the area was strongly affected by various environmental factors including status, soil characteristics, topography and altitude.

## Introduction

Absolutely, environmental gradients play a crucial role in shaping vegetation patterns. The complex interplay between abiotic factors and plants communities is evident across various ecosystems and scales Aljasmi et al.^1^. While strides have been made in understanding of these relationships, there is still a gap in comprehending microclimatic influences on plant abundance in some tropical and subtropical ecosystems Mehmood et al.^2^; Zeb et al.^3^. Bridging the gap between biotic, abiotic components is essential for a comprehensive understanding of ecosystem. The ecological vigor of plant species is often observed in their natural habitats, reflecting adaptation to specific environmental conditions. Distinct compositional groups vary along ecological gradients, showcasing the dynamic nature of the habitats. Fluctuations in species composition along ecological gradients have been longstanding focus of study Mumshad et al.^4^.

Major plant groups of an area are seen as co-evolved populations forming discernible units which could be explored using robust multivariate statistical analysis. Several statistical tools aid ecologists in analyzing large environmental and vegetation datasets, simplifying complexity and revealing patterns. While widely used, their application remains relatively rare in some of the areas, aiding in distinguishing patterns between species and their environments Dar et al.^5^.

Understanding of how site-specific factors influence vegetation diversity and abundance across different ecological habitats, multivariate analysis particularly cluster analysis (CA) and two way cluster analysis (TWCA) allow us to explore complex relationships by incorporating multiple predictors and response variables. The overarching aim is to contribute to a comprehension of the interconnected influence shaping the ecosystem structure and functioning Khan et al.^6^; Khan et al.^7^. Plant species are found in diverse range of environments forming different major plant groups driven by the different ecological factors Rahman et al.^8^.

The interaction between vegetation and environmental gradients is crucial. Studying of plant groups, considering factors like topography, soil, and status provides insights into the intricate relationships in shaping ecological communities Abbas et al.^9^. Comprehensive analysis at both species and major plant group levels is essential for understanding the dynamic interplay between flora and their surroundings Ullah et al.^10^. Phytosociology focuses on the analysis of major plant groups, their characteristics, classification and the impact of environmental factors on their properties and functions Akhlaq et al.^11^. Understanding vegetation distribution pattern and their relationship with environmental factors is important, considering both abiotic conditions and ecosystem processes. Various factors such as topography, soil properties and status play role in influencing plant species richness at different scales. This knowledge helps in comprehending the effects of natural and human induced disturbance on individual species and communities Ullah et al.^10^. The current multivariate analyses of the herbaceous vegetation were conducted with the aim to study the distribution of species in the face of prevailing environmental conditions.

## Materials and methods

### Study area

**Figure 1:**
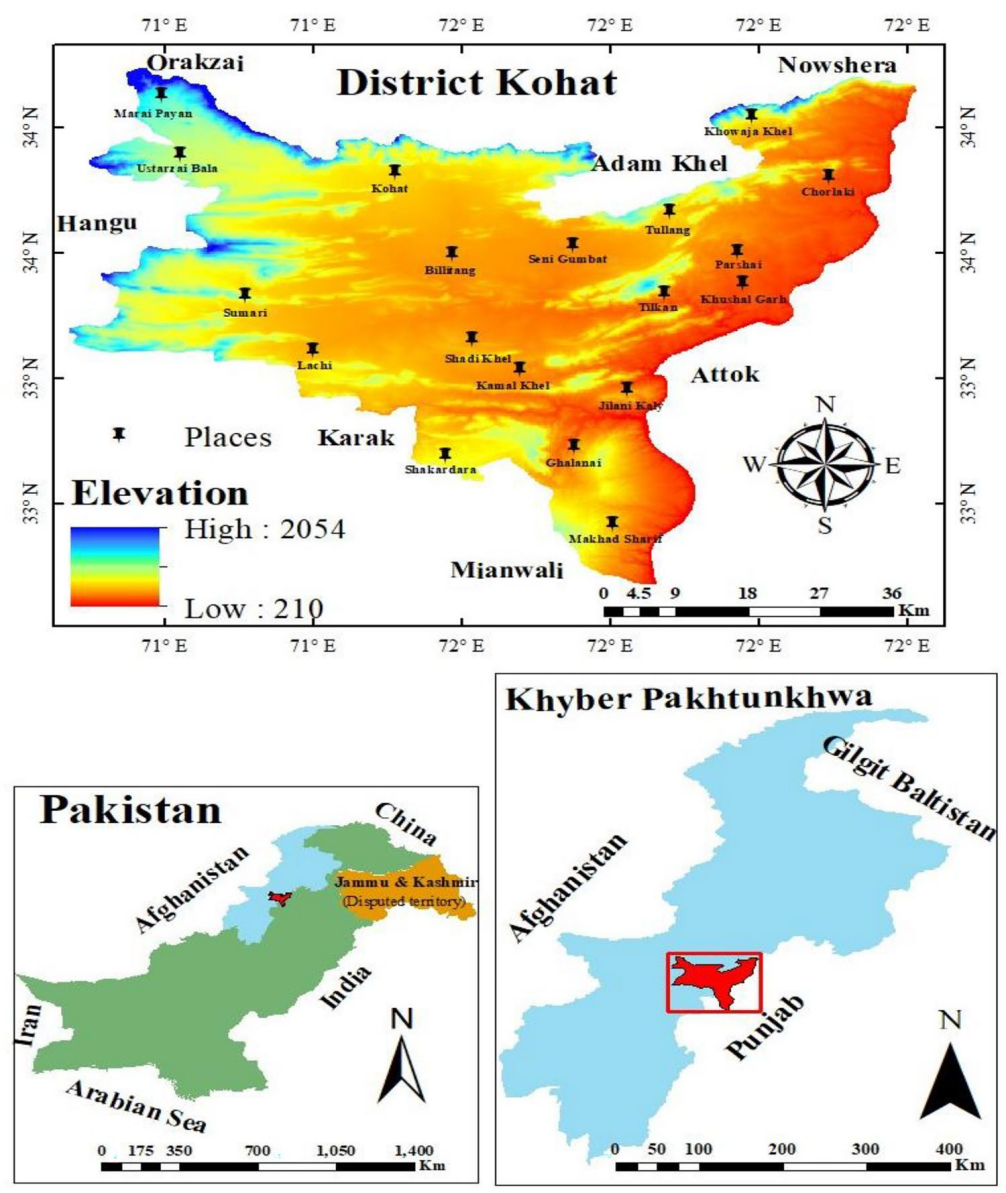
Locational map of the study area with elevation (ArcGIS 10.8.2., https://www.esri.com/en-us/home).

Kohat is a district in the Kohat Division of Khyber Pakhtunkhwa (KP) and is located in its southern part. The research area extends from 33.0676° N to 33.7405° N latitude and from 71.0663° E to 72.0084° E longitude. The total area of the district is about 2991 Km^2^ (Fig. 1). It is bounded on the north by district Peshawar, on the north-west by district Orakzai, on the south by district Karak and district Mianwali, to the west by district Hangu, to the east by River Indus and district Attock and to the north-east by district Nowshera Haseeb et al.^12^. District Kohat climatically falls in the sub-tropical region with slightly arid conditions. Topographically the district is composed of plains and low hills. Most of the area is non-cultivated, comprises of forested areas and rangelands. The cultivated area is mostly non-irrigated. The irrigated area is supplied with the water of rain fed dams. In most of the area the weather is hot from May to September while it is pleasant from October to February. Kohat as a diverse ecological region, harbouring a variety of ecological habitats, having a diverse species composition with different communities, the area direly needs to be studied to fill the scientific knowledge gap.

### Study design

A vegetation survey of the herbaceous layer was conducted during the summer season of 2022–2023 using the Quadrat method. The data was recorded from 40 different sites of the area (Table 1). The herbaceous plant species collected from study area were identified with the help of flora of Pakistan. To get precise image of vegetation of whole district a combination of systematic and random quadrat was used. Quadrats size 1 × 1 m was used for getting vegetational data of the plant species following the minimal area roles. A total of 10 quadrats were applied in each site. These sites were selected on the basis of topography, altitude, status, soil composition and species composition.

**Table 1:** Name, locality and habitat type of the studied forty study sites.

Site name	Abbr	Site name	Abbr
Perennial stream at Torsum	S1	Non-protected rangelands at Ziaratsheikh	S21
Perennial stream Toi	S2	Non-protected rangelands at Krapa	S22
Perennial stream at Nasratkhel	S3	Non-protected rangelands at Merai Payan	S23
Seasonal streams at Tanda	S4	Non-protected rangelands Shadikhel	S24
Seasonal streams at Doda	S5	Protected rangelands of kotal Park	S25
Tanda Dam banks	S6	Protected rangelands of Tanda dam Park	S26
Wetlands	S7	Protected rangelands of Togh Park	S27
Irrigated lands of Tanda	S8	South aspect of Merai high Hills	S28
Irrigated lands of Bana	S9	South aspect of Badasum Hills	S29
Irrigated lands of Doda	S10	South aspect of Kotal Hills	S30
Irrigated lands Gulhasan Banda	S11	South aspect of Merai low Hills	S31
Irrigated lands of Savo	S12	South aspect of Shakardara Hills	S32
Non-irrigated lands of khushalgarh	S13	South aspect of Smari Bala Hills	S33
Non-irrigated lands of Lokhari	S14	South aspect of Tulang Hills	S34
Non-irrigated lands of Darmalak	S15	South aspect of Zameerdam	S35
Non-irrigated lands of Paka	S16	North aspect of Ghorzai Hills	S36
Non-irrigated lands of Smari	S17	North aspect of Tanda Hills	S37
Non-irrigated lands of Sudal	S18	North aspect of Shakardara Hills	S38
Non-irrigated lands of Tulang	S19	North aspect of Tulang Hills	S39
Non-irrigated lands of Zyara	S20	North aspect of Zameerdam Hills	S40

### Soil analysis

A composite soil sample was collected from each site (forty soil samples) and provided to Agriculture Research Institute Tarnab, Peshawar for the physicochemical analysis for understanding its impact on vegetation structure.

### Data analysis

MS excel 2017 was used for basic calculation like frequency, density, cover and relative values. Modern statistical packages PC-ORD version 5 and CANOCO version 5.1 were used to analysis the data. Importance value (IV) was obtained by adding the values of relative density (RD), relative cover (RC) and relative frequency (RF) and all species were sorted by IVs. Plant IV data were treated in CANOCO version 5.1 to measure the environmental gradients responsible for distribution of plants species and identification of plants communities of the area. In present study different multivariate methods like CA, TWCA and detrended correspondence analysis (DCA) etc. were used for identification of plant communities. Names were assigned to the plant communities according to the top three dominant plant species. DCA is a type of indirect gradient and canonical correspondence analysis (CCA) is a type direct gradient analysis to confirm the results of CA and TWCA.

### IUCN policy statement

The collection of plant material complies with relevant institutional, national, and international guidelines and legislation.

## Results

### Floristic diversity

The present vegetation survey of the herbaceous vegetation was conducted during the summer season and was blessed with a rich floristic diversity of a total 216 herbaceous species recorded from forty different sites of the research area.

### Classification of the summer herbaceous vegetation

#### Species area curve

**Figure 2:**
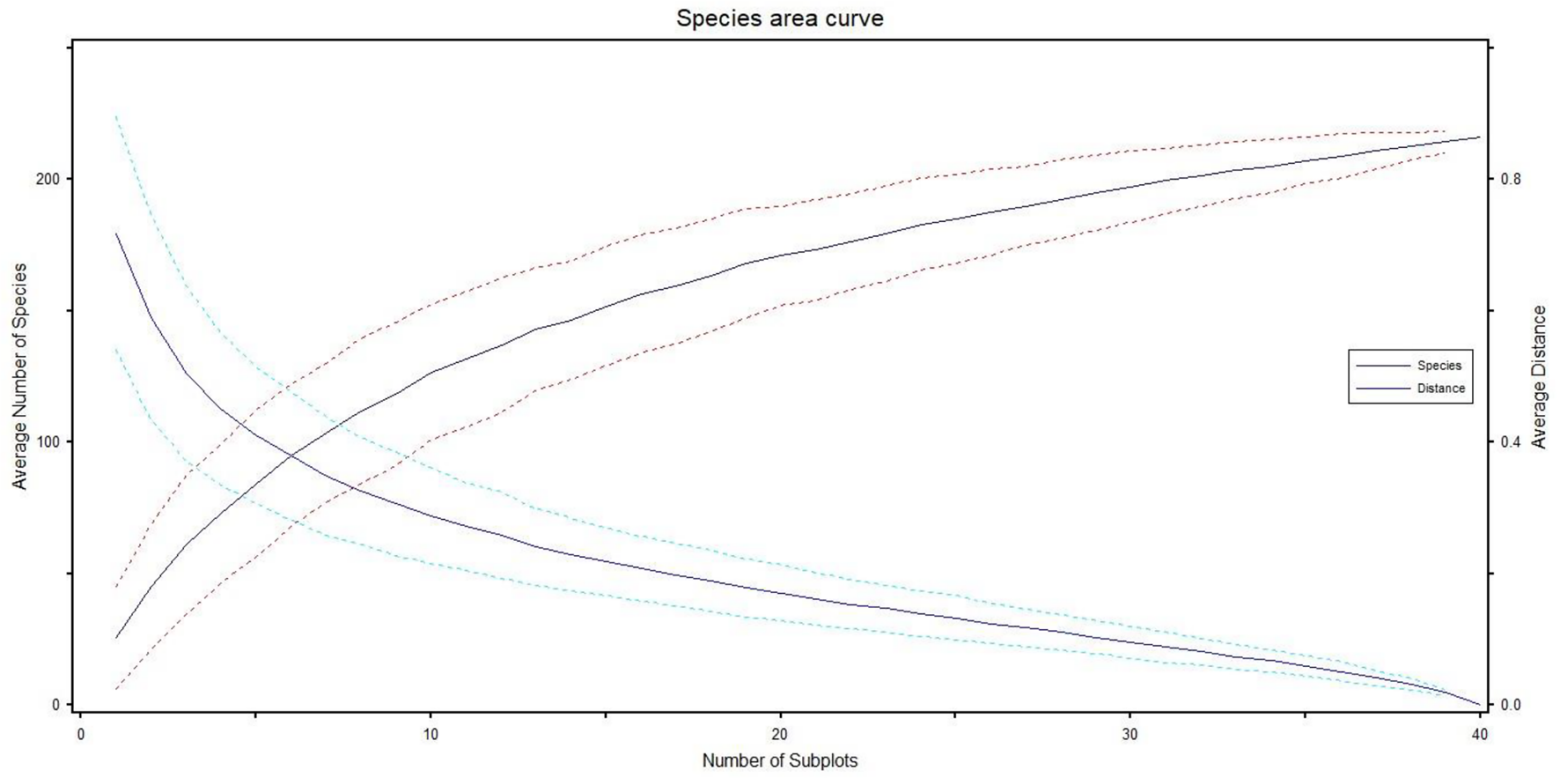
Species area and compositional curves based on IV data and for all 216 species and 40 sampling sites.

Species area curve showed the adequacy of the number of sampling sites in the area. As the graph showing species increase with the increase in number of sampling sites. The results revealed that site 5 showed maximum number of species which were continued up to site 30. Moving further from site 30, decline in species number started and site 40 had the minimum number of species showing adequacy of sampling in the area (Fig. 2).

#### Cluster analysis

**Figure 3:**
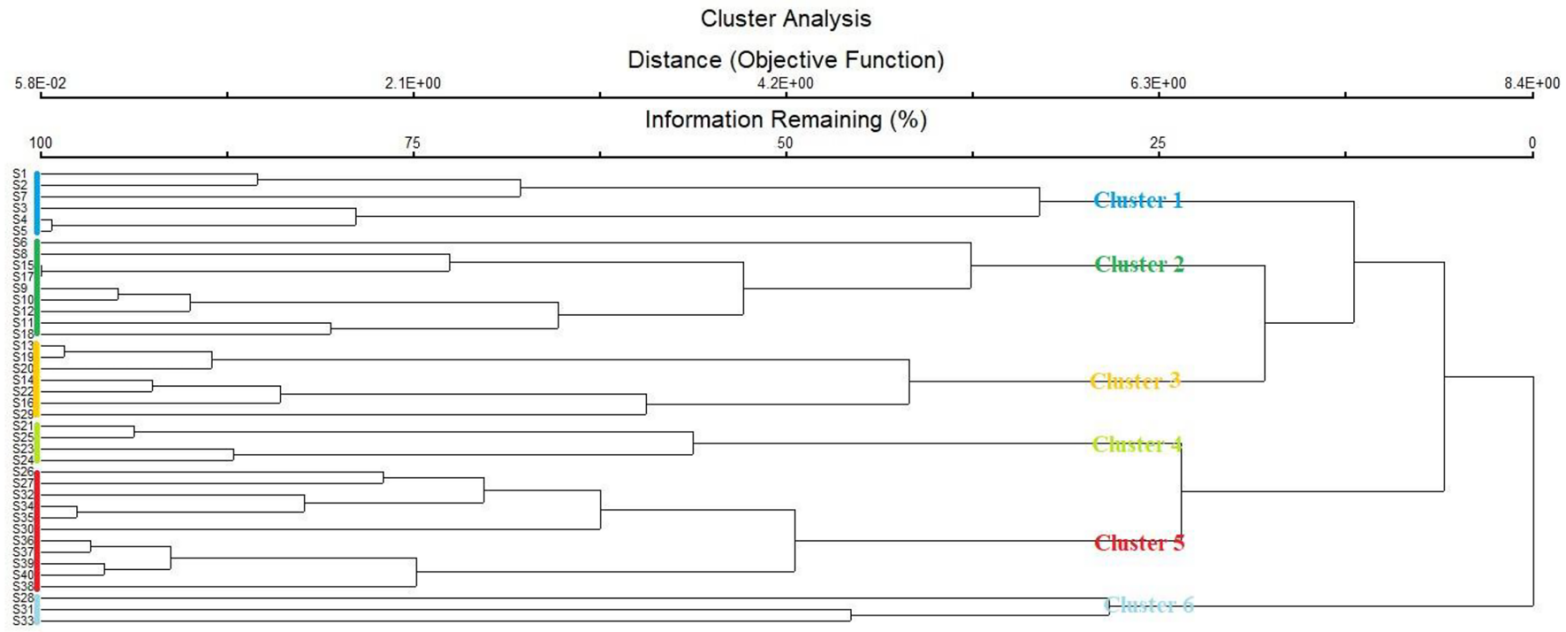
Cluster dendrogram showing all the 40 sites classified into 6 cluster based on Sorenson measures.

The Cluster Analysis (CA) of PC-ORD v.5 classified all the 40 sites into six major plant groups (clusters) on the basis floristic similarity. The detail description of each group is given in (Fig. 3).

#### Two-way cluster analysis

**Figure 4:**
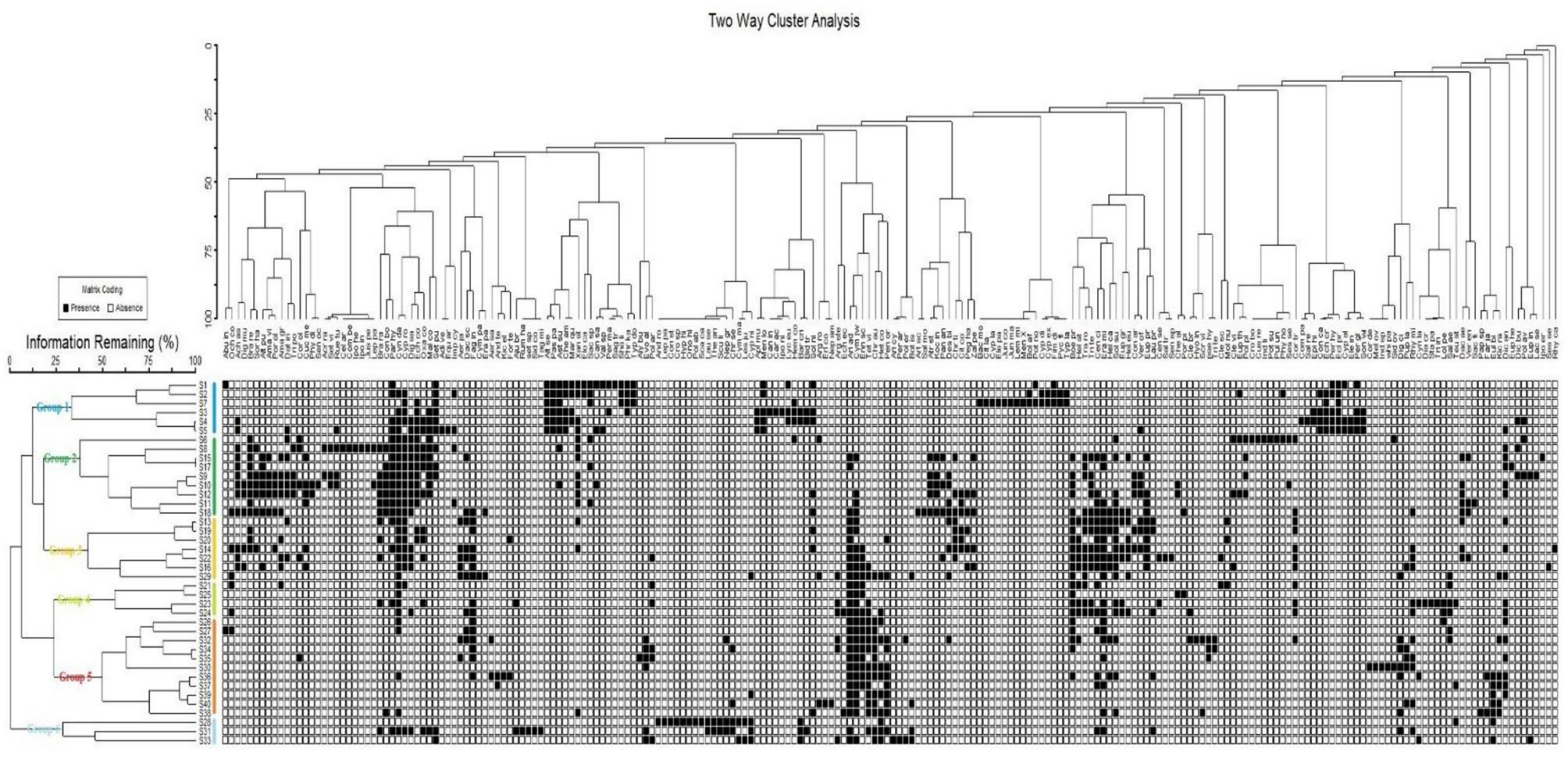
Two-way cluster dendrogram generated through PC-ORD Version 5.1 based on Sorenson measures, showing the distribution of 216 summer herbaceous species in 40 sites and 6 plant groups.

The two-way cluster analysis (TWCA) classified the vegetation data of 216 species recorded at forty different sites of the area mainly into six different major plant groups. The groups were named after the dominant species of each group based on IV (Fig. 4).

##### Group—1 (*Paspalumpaspalodes*, *Alternantherasessilis*, *Typhadomingensis*)

This group was established at the stream beds and wetland habitats where sufficient moisture is available. These sites were located in the plain areas and the altitude of these areas were ranging from 330 to 570 m. The soil were having with clay % particles in the range of (11), silt % (44–76), sand % (13–45), pH (7.9–8.3), Electrical conductivity dsm^−1^ (EC) (0.08–0.22), total soluble salts (TSS) (0.025–0.07), organic matter % (OM) (2–3.08), CaCO_3_% (6.5–9.25)_,_ Soil moisture content % (MC) (25.5–47.2), Nitrogen ppm (N) (0.1–154), Phosphorus ppm (P) (2.6–12.9), Potassium ppm (K) (68–240), This group includes 6 different sites of the area. A total of 72 species were included in this group. The dominant species of the group one with highest importance value (IV) were *Paspalum paspalodes* (193.7) followed by *Alternanthera sessilis* (121.3) and *Typha domingensis* (99.2). The co-dominant species of this group were *Bolboschoenus affinis* (74.2) *Echinochloa crus-galli* (70) *Aster subulatus* (68.3), *Persicaria hydropiper* (65.6), *Saccharum spontaneum* (63.7) and *Apluda mutica* (63.6) (Table 2).

**Table 2:** Importance value of the species in the research area, six major groups clustered with CA and TWCA classification analysis.

Species name	Abbr	Group 1	Group 2	Group 3	Group 4	Group 5	Group 6
*Abutilon indicum* (L.) Sweet	Abu in	3	0	0	0	7.678806	0
*Achyranthus aspera* L	Ach as	11	71.01421	17.71094	0	0	0
*Acrachne racemosa* (Heyne ex Roem. & Schult.) Ohwi	Acr ra	0	7.640325	0	0	0	0
*Adiantum capillus-veneris* L	Adi ve	4	0	0	0	0	0
*Aerva javanica* (Burm. f.) Schult	Aer ja	0	0	49.64393	0	16.46995	0
*Aerva sanguinolenta* (L.) Blume	Aer sa	0	0	0	0	20.75945	0
*Ajuga bracteosa* Wall. ex Benth	Aju br	0	0	0	8.013526	0	7.186743
*Alternanthera pungens* Kunth	Alt pu	0	32.11331	5.743649	0	0	0
*Alternanthera sessilis* (L.) R. Br. ex DC	Alt se	**121**	0	0	0	0	0
*Alysicarpus bupleurifolius* (L.) DC	Aly bu	0	0	0	0	6.067397	0
*Amaranthus graecizans* L	Ama gr	0	21.62944	15.20855	3.991971	0	0
*Amaranthus viridis* L	Ama vi	0	15.61606	0	0	0	0
*Andrachne telephiodes* L	And te	0	0	0	0	12.34755	0
*Androsace rotundifolia* Hardwicke	And ro	0	0	0	0	0	7.741767
*Apluda mutica* L	Apl mu	64	0	0	0	0	0
*Argyrolobium roseum* (Camb.) Jaub	Arg ro	0	3.844738	2.270739	0	5.4541	0
*Argyrolobium stenophyllum* Boiss	Arg ste	0	0	2.506033	11.97655	6.477791	2.413818
*Aristida adscensionis* L	Ari ad	0	33.55592	56.97838	**96.87644**	**383.4593**	22.75793
*Aristida cyanantha* Nees. ex Steud	Ari cy	0	0	0	0	0	32.56335
*Artemisia scoparia* Waldst. & Kit	Art sc	0	10.20863	0	0	0	0
*Leptorhabdos parviflora* (Benth.) Benth	Lep pa	0	0	0	0	0	3.659467
*Aster subulatus* Michaux	Ast su	68	6.235452	0	0	0	0
*Atriplex stocksii* Boiss	Atr st	0	40.78349	0	0	0	0
*Bacopa monnieri* (L.) Pennell	Bac mo	18	0	0	0	0	0
*Barleria cristata* L	Bar cri	25	0	0	0	0	13.47311
*Bidens tripartita* L	Bid tr	25	0	0	0	0	4.774591
*Boerhavia procumbens* Banks. ex Roxb	Boe pr	0	26.32068	86.85247	**62.90297**	64.65788	0
*Bolboschoenus affinis* (Roth.) Droboy	Bol af	74	0	0	0	0	0
*Bothrriochloa ischaemum* (L.) Keng	Bot is	0	0	6.24736	0	24.4025	**68.65145**
*Brachiaria ramosa* (L.) Stapf	Bra ra	0	64.0772	26.46813	3.946154	0	5.082037
*Brachiaria reptans* (L.) Gardner & Hubbard	Bra re	0	**148.4236**	23.46135	0	0	0
*Canna indica* L	Can in	21	0	0	0	0	0
*Cannabis sativa* L	Can sa	21	4.857561	0	0	0	0
*Carex acutiformis* Ehrh	Car ac	5	0	0	0	0	0
*Celosia argentea* L	Cel ar	0	5.274493	0	0	0	0
*Cenchrus ciliaris* L	Cen ci	0	30.45637	74.39292	36.03411	20.43588	2.412153
*Cenchrus setigerus* Vahl	Cen se	0	0	11.75622	8.79846	0	0
*Chenopodium album* L	Che al	0	6.746659	0	24.64948	0	0
*Chenopodium ambrosoides* L	Che am	25	0	0	0	0	0
*Chrozophora tinctoria* (L.) Raffin	Chr ti	0	14.05837	43.53683	0	0	0
*Chrysopogon aucheri* (Boiss) Stapf	Chr au	0	0	8.423831	0	240.1184	52.68717
*Chrysopogon serrulatus* Trin	Chr se	0	0	0	0	34.84841	**59.69002**
*Citrullus colocynthis* (L.) Schard	Cit co	0	10.9593	13.23474	0	0	0
*Citrullus lanatus* (Thunb.) Mats. & Nakai	Cit la	3	0	0	0	0	0
*Cleome brchycarpa* Vahl. ex DC	Cle br	0	0	0	4.784462	9.502963	0
*Cleome scaposa* DC	Cle sc	0	0	6.740083	20.02709	0	0
*Cleome viscosa* L	Cle vi	0	17.0715	6.468286	0	0	0
*Commelina benghalensis* L	Com be	0	12.32648	0	0	0	0
*Commelina paludosa* Blume	Com pa	4	0	0	0	0	0
*Convolvulus arvensis* L	Con ar	0	7.606893	11.01419	0	0	0
*Conyza bonariensis* (L.) Cronquist	Con bo	0	19.69541	0	0	4.595865	0
*Conyza canadensis* (L.) Cronquist	Con ca	21	0	0	0	0	0
*Conyza stricta* Willd	Con st	0	0	0	0	0	7.741767
*Corbichonia decumbens* (Forssk.) Exell	Cor de	0	0	0	0	6.579299	0
*Corchorus olitorius* L	Cor ol	0	18.27782	1.611406	0	5.270584	0
*Corchorus tridens* L	Cor tr	0	31.97648	36.20554	13.31724	9.611565	0
*Crotolaria sp*	Cro sp	0	0	0	0	0	5.915046
*Croton bonplandianus* Baill	Cro bo	0	10.62208	0	0	0	0
*Cucumis melo* L. subsp. *Agrestis* (Naudin) Pangalo	Cuc me	0	25.2057	5.533562	0	0	0
*Cuscuta reflexa* Roxb	Cus re	0	7.909257	0	0	0	0
*Cymbopogon jwarancusa* (Jones) Schult	Cym jw	0	52.65668	**121.3099**	**417.973**	**632.449**	3.990531
*Cymbopogon martini* (Roxb.) Wats	Cym ma	0	0	0	0	0	45.25781
*Cymbopogon pospischilii* (Schum.) Hubbard	Cym pa	0	0	4.682504	0	0	0
*Cynodon dactylon* (L.) Pers	Cyn da	39	**305.2475**	**237.8871**	47.29015	22.09343	7.236458
*Cynoglossum lanceolatum* Forssk	Cyn la	0	0	0	6.372307	2.314896	4.457265
*Cyperus alopecuroides* Rottb	Cyp al	16	0	0	0	0	0
*Cyperus difformis* L	Cyp di	8	0	0	0	0	0
*Cyperus exaltatus* Retz	Cyp ex	15	0	0	0	0	0
*Cyperus laevigatus* L	Cyp la	4	0	0	0	0	0
*Cyperus niveus* Retz	Cyp ni	0	0	2.506033	0	12.54979	43.93268
*Cyperus rotundus* L	Cyp ro	15	142.3392	106.1682	7.160897	0	7.35191
*Dactyloctenium aegyptium* (L.) Willd	Dac ae	0	36.37724	21.77448	0	0	0
*Dactyloctenium scindicum* Boiss	Dac sc	0	6.399909	75.01991	21.75565	9.522816	0
*Datura innoxia* Miller	Dat in	3	36.88827	7.315852	0	0	0
Papilionaceae	Pap sp	0	0	0	0	4.595865	0
*Desmostachya bippinata* (L.) Stapf	Des bi	0	65.3511	18.37877	0	4.44753	0
*Dianthus crinitus* Sm	Dia cr	0	0	0	6.472328	0	0
*Dichanthium annulatum* (Forssk.) Stapf	Dic an	3	59.51926	23.13415	16.60495	45.87666	0
*Dicliptera bupleuroides* Nees	Dic bu	2	5.541362	0	0	0	0
*Digera muricata* (L.) Mart	Dig mu	0	13.4578	7.92876	0	0	0
*Digitaria ciliaris* (Retz.) Koel.p	Dig ci	0	0	4.682504	0	93.73566	5.547786
*Digiteria sanguinalis* (L.) Scop	Dig sa	14	77.44747	15.26632	5.148929	0	2.669885
*Echinochloa colona* (L.) Link	Ech co	25	83.5719	11.31272	0	0	0
*Echinochloa crus-galli* (L.) P. Beauv	Ech cr	70	0	0	0	0	0
*Echinops echinatus* Roxb	Ech ec	0	0	0	4.260902	2.818754	0
*Eclipta prostrata* L	Ecl pr	29	0	0	0	0	0
*Eleocharis palustris* (L.) Roem & Schult	Ele pa	11	0	0	0	0	0
*Eleusine indica* (L.) Gaertn	Ele in	18	0	0	0	0	0
*Elodea canadensis* Michx	Elo ca	5	0	0	0	0	0
*Enneapogon schimperanus* (Hochst. ex A. Rich.) Renv	Enn sc	0	0	14.66266	41.137	111.6718	0
*Epilobium hirsutum* L	Epi hi	13	0	0	0	0	0
*Equisetum arvense* L	Equ ar	2	0	0	0	0	0
*Eragrostis minor* Host	Era mi	0	20.63204	**155.4153**	11.03555	59.27197	22.33343
*Eragrostis papposa* (Roem. & Schult.) Steud	Era pa	0	3.928638	4.682504	0	0	0
*Erioscirpus comosus* (Wall.) Palla	Eri co	0	0	0	0	6.459125	0
*Eulaliopsis binata* (Retz.) C. E. Hubbard	Eul bi	0	0	0	0	156.2419	10.27523
*Euphorbia granulata* Forssk	Eup gr	0	1.848875	27.77685	9.429791	0	0
*Euphorbia heterophylla* L	Eup he	0	4.661975	0	0	0	0
*Euphorbia indica* Lam	Eup in	0	3.396094	0	0	0	2.228633
*Euphorbia prostrata* Ait	Eup pr	0	15.25118	55.62572	0	0	0
*Euphorbia thymifolia* L	Eup th	2	9.365175	0	0	0	0
*Evolvulus alsinoides* (L.) L	Evo al	0	0	0	0	70.62798	15.74718
*Fagonia indica* Burm. f	Fag in	0	1.691146	50.45182	17.44087	65.56056	0
*Farsetia jacquemontii* Hook. f. & Thoms	Far ja	0	0	0	0	8.517208	0
*Fimbristylis dichotoma* (L.) Vahl	Fim di	22	0	0	0	0	0
*Forsskaolea tenacissima* L	For te	0	0	4.185372	0	4.800464	0
*Heliotropium calcareum* Stocks	Hel ca	0	3.77091	14.89433	0	6.982207	0
*Heliotropium europaeum* L	Hel eu	0	3.616328	27.16176	9.568925	0	0
*Heliotropium strigosum* Willd	Hel st	0	0	30.90373	17.66245	4.44753	0
*Hemarthria compressa* (L.f.) R. Br	Hem co	25	11.754	0	0	0	0
*Heteropogon contortus* (L.) Beauv	Het co	0	0	3.976621	4.784462	143.9775	**76.40699**
*Hyparrhenia hirta* (L.) Stapf	Hyp hi	0	0	0	0	0	3.870883
*Hyoscyamus insanus* Stocks	Hyo in	0	0	0	0	4.44753	0
*Impereta cylindrica* (L.) Raeush	Imp cy	11	11.01545	0	0	0	0
*Indigofera linifolia* (Linn. f) Retz	Ind li	0	18.45588	0	0	0	0
*Ipomoea eriocarpa* R. Br	Ipo er	0	3.102185	0	0	0	0
*Ipomoea hederacea* Jacq	Ipo he	0	5.274493	0	0	0	0
*Ipomoea indica* (Burm. f.) Merrill	Ipo in	0	5.274493	0	0	0	0
*Ipomoea nil* (L.) Roth	Ipo ni	5	0	0	0	0	0
*Juncus compressus* Jacq	Jun co	4	0	0	0	0	0
*Juncus maritimus* Lam	Jun ma	7	0	0	0	0	0
*Justicia peploides* T. Anders	Jus pe	0	5.653651	0	0	0	0
*Kickxia elatine* (L.) Dumort	Kic el	0	0	0	0	2.534676	0
*Kickxia ramosissima* (Wall.) Janch	Kic ra	0	0	3.976621	0	57.59962	0
*Kochia indica* Wight	Koc in	0	24.87182	0	0	0	0
*Lactuca serriola* L	Lac se	0	3.629194	0	0	0	0
*Salvia reflexa* Hornem	Sal re	4	0	0	0	0	0
*Pervoskia arobetenoides* Karel	Per ar	0	0	0	0	0	10.95044
*Launea procumbens* Roxb	Lau pr	0	0	16.95126	2.574464	7.164469	4.259127
*Launea secunda* Hook. f	Lau se	0	0	0	0	0	17.77235
*Lemna minor* L	Lem mi	14	0	0	0	0	0
*Leptochloa panicea* (Retz.) Ohwi	Lep pa	0	12.84087	0	0	0	0
*Lespedeza juncea* (Linn. f.) Pers	Les ju	0	0	0	0	0	28.3071
*Lolium perenne* L	Lol pe	0	0	0	33.18525	9.904893	0
*Lycopus europaeus* L	Lyc eu	40	0	0	0	0	0
*Melhania ovata* (Cay.) Spreng	Mel ov	0	0	0	0	5.005778	0
*Malvastrum coromandelianum* (L.) Garcke	Mal co	23	84.74115	0	0	0	13.85802
*Marsilea quadrifolia* L	Mar qu	29	0	0	0	0	0
*Mentha longifolia* (L.) Huds	Men lo	58	0	0	0	0	0
*Mentha X piperita* L	Men X	4	0	0	0	0	0
*Mollugo nudicaulis* Lam	Mol nu	0	0	15.00543	10.39332	0	0
*Nepeta griffithii* Hedge	Nep gr	0	0	0	0	15.4589	37.76319
*Nepeta amicorum* Rech. F	Nep am	0	0	0	0	5.4541	0
*Ochthochloa compressa* (Forssk.) Hilu	Och co	0	3.297724	8.064181	25.60638	59.73592	0
*Onosma hispida* Wall. ex G. Don	Ono hi	0	0	0	0	0	3.870883
*Oxalis corniculata* L	Oxa co	10	29.96191	10.39937	8.334786	0	4.259127
*Panicum antidotale* Retz	Pan an	0	27.08744	5.598731	0	0	0
*Parthenium hysterophorus* L	Par hy	39	**235.9212**	12.32057	3.946154	0	28.43953
*Paspalum paspalodes* (Michx.)Scribner	Pas pa	**194**	0	0	0	0	0
*Peganum harmala* L	Peg ha	0	22.97494	21.76099	0	0	0
*Pennisetum orientale* L. C. Rich	Pen or	0	0	104.9595	0	**267.7623**	2.412153
*Persicaria glabra* (Willd.) M. Gomes	Per gl	35	0	0	0	0	0
*Persicaria hydropiper* (L.) Delabre	Per hy	66	0	0	0	0	0
*Persicaria maculosa* Gray	Per ma	18	0	0	0	0	0
*Phragmites karka* (Retz.) Trin. ex Steud	Phr ka	41	0	0	0	0	0
*Phyla nodiflora* (L.) Greene	Phy no	4	27.64797	0	0	0	0
*Physalis divaricata* D. Don	Phy di	0	18.03961	0	0	0	0
*Indigofera sp*.	Ind sp	0	0	0	0	16.67583	0
*Polygala abyssinica* R.r. ex.Fresen	Pol ab	0	0	0	0	0	4.511158
*Polygala arvensis* Willd	Pol ar	0	0	6.945844	16.0426	42.93509	13.55698
*Polygala erioptera* DC	Pol er	0	0	0	0	2.278066	4.457265
*Polygonum aviculare* L	Pol av	4	17.6134	0	0	0	0
*Polygonum plebjum* R. Br	Pol pl	0	19.22223	0	0	0	0
*Portulaca oleracea* L	Por ol	0	9.893264	2.044286	0	0	0
*Portulaca pilosa* L	Por pi	0	3.613181	0	12.90214	0	0
*Potamogeton nodosus* Poiret	Pot no	6	0	0	0	0	0
*Potentilla supina* L	Pot su	0	2.273267	0	0	0	0
*Sesuvium sesuvioides* (Fenzl) Verdc	Ses se	0	0	8.066826	0	0	0
*Pseudgaillonia hymenostephana* (Daub. & Spach) Lincz	pse hy	0	0	0	0	52.11251	0
*Pulicaria undulata* (L.) Mey	Pul un	0	33.17549	0	0	0	0
*Pupalia lappaceae* (L.) Juss	Pup la	0	0	14.29357	0	51.74986	0
*Pycreus flavidus* T. Koyama	Pyc fl	23	0	0	0	0	0
*Rhynchosia capitata* (Heyne ex Roth.) DC	Rhy ca	0	0	12.85242	0	0	0
*Rhynchosia minima* (L.) DC	Rhy mi	0	2.982266	26.0119	13.21276	32.87718	5.290055
*Ruellia tuberosa* L	Rue tu	7	37.13021	0	0	0	0
*Rumex hastatus* D. Don	Rum ha	0	0	0	0	0	10.83784
*Saccharum filifolium* Steud	Sac fi	0	7.489462	0	0	0	0
*Saccharum spontaneum* L	Sac sp	64	51.0026	0	0	0	0
*Sagittaria trifolia* L	Sag tr	8	0	0	0	0	0
*Salsola tragus* L	Sal tr	0	0	3.16428	0	0	0
*Salvia aegyptiaca* L	Sal ae	0	0	4.541478	31.01271	6.762731	0
*Salvia moocroftiana* Wall. ex Benth	Sal mo	0	3.613181	0	0	0	0
*Scabiosa candollei* DC	Sca ca	0	0	0	0	0	2.255579
*Scrozonera virgata* DC	Scr vi	0	0	0	0	8.764844	0
*Scutellaria linearis* Benth	Scu li	0	0	0	0	0	20.59435
*Senna occidentalis* (L.) Link	Sen oc	0	4.857561	0	0	0	0
*Senna sp*	Sen sp	0	0	3.16428	0	0	0
*Sesbania sesban* (L.) Merrill	Ses se	0	8.669898	0	0	0	0
*Setaria pumila* (Poir.) Roem. & Schult	Set pu	40	16.71255	0	0	0	25.26339
*Setaria viridis* (L.) P. Beauv	Set vi	0	26.57803	0	0	0	0
*Seteria sp*	set sp	0	0	0	0	0	17.60169
*Shoenoplectus litoralis* (Schard.) Palla	Sho li	33	0	0	0	0	0
*Sida cordifolia* L	Sid co	0	0	0	0	0	4.259127
*Sida ovata* Forssk	Sid ov	0	4.723859	0	0	4.611478	0
*Solanum nigrum* L	Sol ni	13	5.079886	4.429093	0	4.595865	0
*Solanum surattense* Burm. f. k	Sol su	0	25.22666	61.09096	10.82674	43.33389	0
*Sonchus wighthianus* DC	Son wi	5	0	0	0	0	0
*Sorghum halepense* (L.) Pers	Sor ha	0	29.73851	7.084209	0	0	0
*Stachys parviflora* Benth	Sta pa	0	0	0	18.26064	0	0
*Tagetes minuta* L	Tag mi	0	0	0	0	0	9.033717
*Tetrapogon villosus* Desf	Tet vi	0	0	36.85479	16.73763	180.4269	4.774591
*Teucrium stocksianum* Boiss	Teu st	0	0	0	17.03322	42.03002	12.25293
*Themeda anathera* (Nees ex Steud.) Hack	The an	0	0	0	0	0	54.26676
*Tragus roxburghii* Panigrahi	Tra ro	0	13.48273	51.72118	22.54304	0	0
*Trianthema portulacastrum* L	Tri po	0	31.72441	0	0	0	0
*Tribulus terrestris* L	Tri te	0	34.08471	43.12175	13.68294	0	0
*Trichodesma indicum* (L.) R. Br	Tri in	0	0	0	4.288611	0	0
*Tricholaena teneriffae* (L.F.) Link	Tri te	0	0	0	0	13.26171	0
*Typha domingensis* Pers	Typ do	**99**	0	0	0	0	0
*Typha latifolia* L	Typ la	12	0	0	0	0	0
*Vallisneria spiralis* L	Val sp	8	0	0	0	0	0
*Verbena officinalis* L	Ver of	3	19.54913	18.63744	0	0	0
*Verbesina encelioides* (Cav.) Benth & Hook. F. ex A. Gray	Ver en	0	25.93516	6.945844	0	0	0
Unknown	whi pa	0	0	0	0	9.383941	0
*Xanthium strumarium* L	Xan st	46	40.45485	19.52191	0	5.431519	0
*Zaleya pentandra* (L.) Jeffrey	Zal pe	0	12.75691	11.35831	0	0	0

The bold values in the table showing the top three IV of the dominant species of the major groups.

##### Group—2 (*Cynodondactylon*, *Partheniumhysterophorus*, *Brachiariaramose*)

Group 2 comprises of 9 sites. This group was established in the cultivated plain habitats which have mainly mesic types of condition. The altitude of these areas were ranging from 405 to 575 m. The soil were having with clay % particles in the range of (10–11), silt % (24–66), sand % (23–65), pH (8.1–8.6), EC dsm^−1^ (0.06–0.17), TSS % (0.019–0.05), OM % (1.03–3.1), CaCO_3_% (7.25–10)_,_ MC% (9.4–19.2), N ppm (0.051–0.155), P ppm (2.6–8), K ppm (84–562), *Cynodon dactylon* was the dominant species of this group having the IV of (305), followed by *Parthenium hysterophorus* (235.9) and *Brachiaria ramosa* (148). While the other prominent species of this group were *Cyperus rotundus* (142), *Malvastrum coromandelianum* (84.7), *Echinochloa colona* (83.5), *Digiteria sanguinalis* (77.4) and *Achyranthus aspera* (71) (Table 2).

##### Group—3 (*Cynodondactylon*, *Eragrostisminor*, *Cymbopogonjwarancusa*)

This group includes 7 sites. This group was established mostly in the non-irrigated plain areas mostly which were having xeric conditions. The altitude of these areas were ranging from 315 to 560 m. The soil were having with clay % particles in the range of (10–11), silt % (14–40), sand % (49–75), pH (8–8.9), EC dsm^−1^ (0.05–0.23), TSS % (0.016–0.073), OM % (0.34–1.72), CaCO_3_% (6.5–9.25)_,_ MC% (8–9.9), N ppm (0.017–0.086), P ppm (2.4–7.4), K ppm (70–212). *Cynodon dactylon* was the dominant species of group 3 having the IV of 237 followed by *Eragrostis minor* (155) and *Cymbopogon jwarancusa* (121). Other co-dominant species of this group were *Cyperus rotundus* (106), *Pennisetum orientale* (104), *Boerhavia procumbens* (86.8), *Dactyloctenium scindicum* (75), *Cenchrus ciliaris* (74.3) and *Solanum surattense* (61) (Table 2).

##### Group—4 (*Cymbopogonjwarancusa*, *Aristidaadscensionis*, *Boerhaviaprocumbens*)

This group was relatively small comprised of 4 sites. These sites were located at the rangeland areas and the altitude of these areas were ranging from 365 to 760 m. The soil were having with clay % particles in the range of (11), silt % (26–58), sand % (31–63), pH (8–8.4), EC dsm^−1^ (0.11–0.16), TSS % (0.035–0.051), OM % (0.34–2.76), CaCO_3_% (6.5–9.25)_,_ MC% (7.8–8.6), N ppm (0.017–0.138), P ppm (3.7–7.4), K ppm (392–510). *Cymbopogon jwarancusa* was the dominant species having the IV of 417 followed by *Aristida adscensionis* (98.8) and *Boerhavia procumbens* (62.9). other prominent species of this community were *Cynodon dactylon* (47.2), *Enneapogon schimperanus* (41.1), *Cenchrus ciliaris* (36), *Lolium perenne* (33.1) and *Salvia aegyptiaca* (31) (Table 2).

##### Group—5 (*Cymbopogonjwarancusa*, *Aristidaadscensionis*, *Pennisetumorientale*)

This group was the largest group among all group. It includes 11 sites. These sites were located mostly at the hilly region of low altitude ranging from 460 to 670 m. The soil were having with clay % particles in the range of (11), silt % (22–52), sand % (37–67), pH (8.2–8.9), EC dsm^−1^ (0.05–0.11), TSS % (0.016–0.035), OM % (0.62–3.1), CaCO_3_% (6.75–9.75)_,_ MC% (7.8–9.9), N ppm (0.031–0.155), P ppm (2.4–8.2), K ppm (70–522). *Cymbopogon jwarancusa* was the dominant species having the IV 632 followed by *Aristida adscensionis* (383) and *Pennisetum orientale* (267). Other prominent members of this group were *Chrysopogon aucheri* (240), *Tetrapogon villosus* (180), *Eulaliopsis binata* (156), *Heteropogon contortus* (143) and *Enneapogon schimperanus* (111) (Table 2).

##### Group—6 (*Heteropogoncontortus*, *Bothrriochloaischaemum*, *Chrysopogonserrulatus*)

This was the smallest group comprised of 3 sites. This group was established at the high-altitude hills. The altitude of these areas were ranging from 895 to 1225 m. The soil were having with clay % particles in the range of (10–11), silt % (19–40), sand % (49–71), pH (8.1–8.9), EC dsm^−1^ (0.11–0.19), TSS % (0.035–0.061), OM % (0.48–2.07), CaCO_3_% (8.25–9.75)_,_ MC% (8.3–9.3), N ppm (0.024–0.103), P ppm (4.6–10.2), K ppm (104–198). The dominant species of this group was *Heteropogon contortus* having IV of (76.4) followed by *Bothrriochloa ischaemum* (68.6) and *Chrysopogon serrulatus* (59.6). Other prominent members of this group were *Themeda anathera* (54.2). *Chrysopogon aucheri* (52.6), *Cymbopogon martini* (45.2) and *Cyperus niveus* (43.9) (Table 2).

### Ordination

#### Detrended correspondence analysis (DCA)

##### DCA ordination of sites

DCA ordination revealed about the pattern in complex data set. The gradient length for axis 1 was 7.62 with the Eigen value of 0.801, for axis 2 gradient length was 3.30 with Eigen value of 0.326, for axis 3 gradient length was 2.53 with Eigen value of 0.22 and for axis 4 the gradient length 2.75 with Eigen value of 0.159. The DCA diagram was used to analyze the ordination of different sites on the basis of species composition. The present DCA diagram of all the stations revealed the position of different station along the axis. Based on the floristic composition data the vegetation of the area was mainly grouped into six major groups. Group 1 comprises of S1, S2, S3 S4, S5, S7 these sites cluster under the influence of high moisture content, group two comprises of S6, S8, S9, S10, S11, S12, S15, S17, S18, group 3 comprises of S13, S14, S16, S19, S20, S22 these sites cluster under the influence of topography and low altitude, group 4 comprises of S21, S23, S24, S25 these were rangelands, group 5 comprises of S26, S27, S30, S32, S34, S35, S36, S37, S38, S39 and S40, these sites were clustered due to the hilly landscape with low altitude while group 6 comprises of S28, S31 and S33 which were cluster under the strong influence of high altitude and south aspect. Among all the station, S6, S28 and S33 located separately because of their different extremes of ecological conditions.

**Figure 5:**
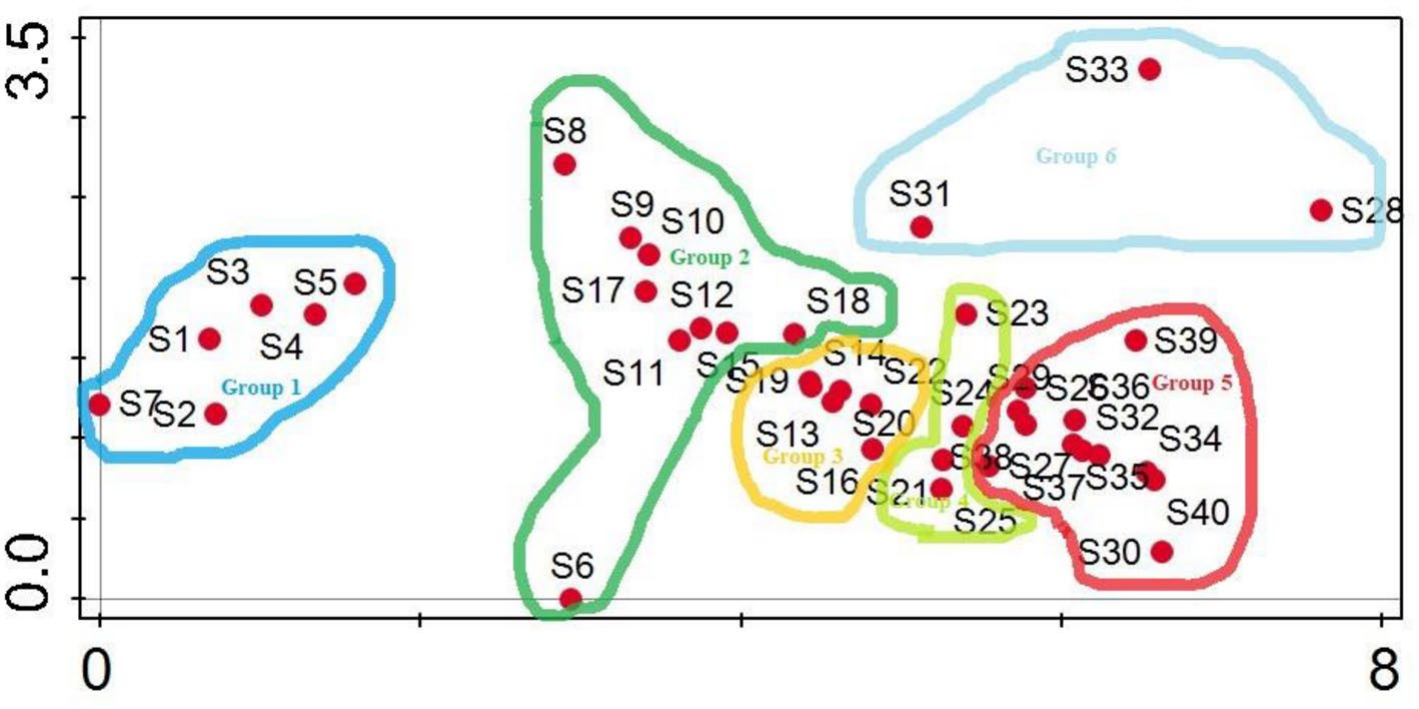
DCA ordination plot of the 40 different sites in the study area.

DCA confirmed the CA classification of the vegetation data. In the present DCA graph, group 1 locate separately at left side, group 2, group 3 and group 4 locate at the mid center of the graph while group 5 and group 6 locate at the right side (Table 3) (Fig. 5).

**Table 3:** Description of the four axes of the DCA analysis.

Axes	1	2	3	4	Total inertia
Eigen value	0.801	0.326	0.221	0.159	7.27
Lengths of gradient	7.62	3.30	2.53	2.75	
Cumulative percentage variance of species data	11.01	15.50	18.54	20.73

##### DCA ordination of the species

All the 216 recorded species were analyzed through DCA analysis. In the graph the species which were lying closed to each other showed strong correlation with each other while the species which were located away and faraway from each other showed no correlation with each other.

**Figure 6:**
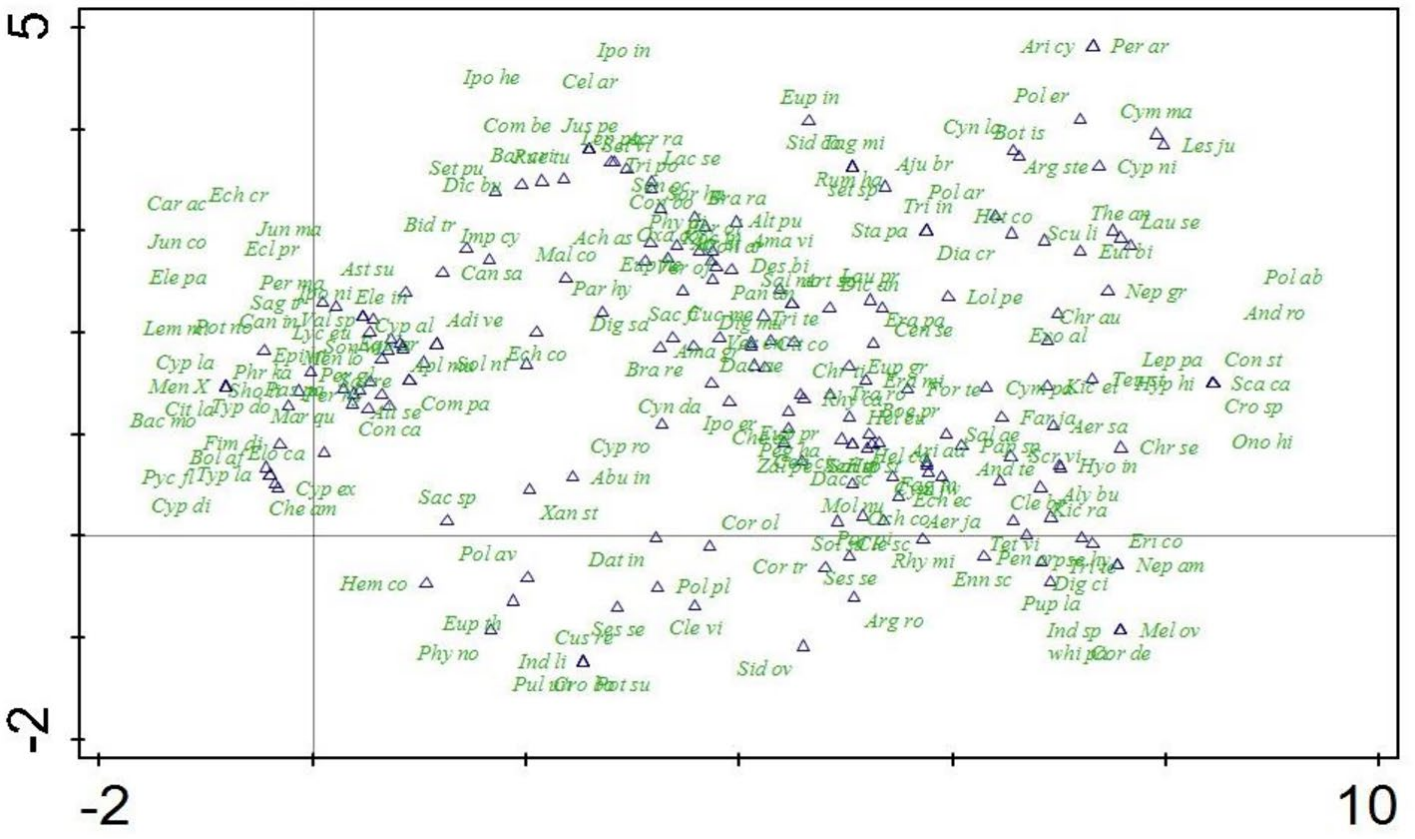
DCA ordination plot presenting the distribution of 216 herbaceous plant species of the area.

The driving ecological factors for the clustering of vegetation into different groups were topography, altitude and soil moisture content. In both the DCA plots from left to right the soil moisture contents decreases while the altitude increases and the topography from left to right changes from plains to hilly landscape (Table 3) (Fig. 6).

### Canonical correspondence analysis (CCA)

#### CCA analysis of the species and environmental factors

**Figure 7:**
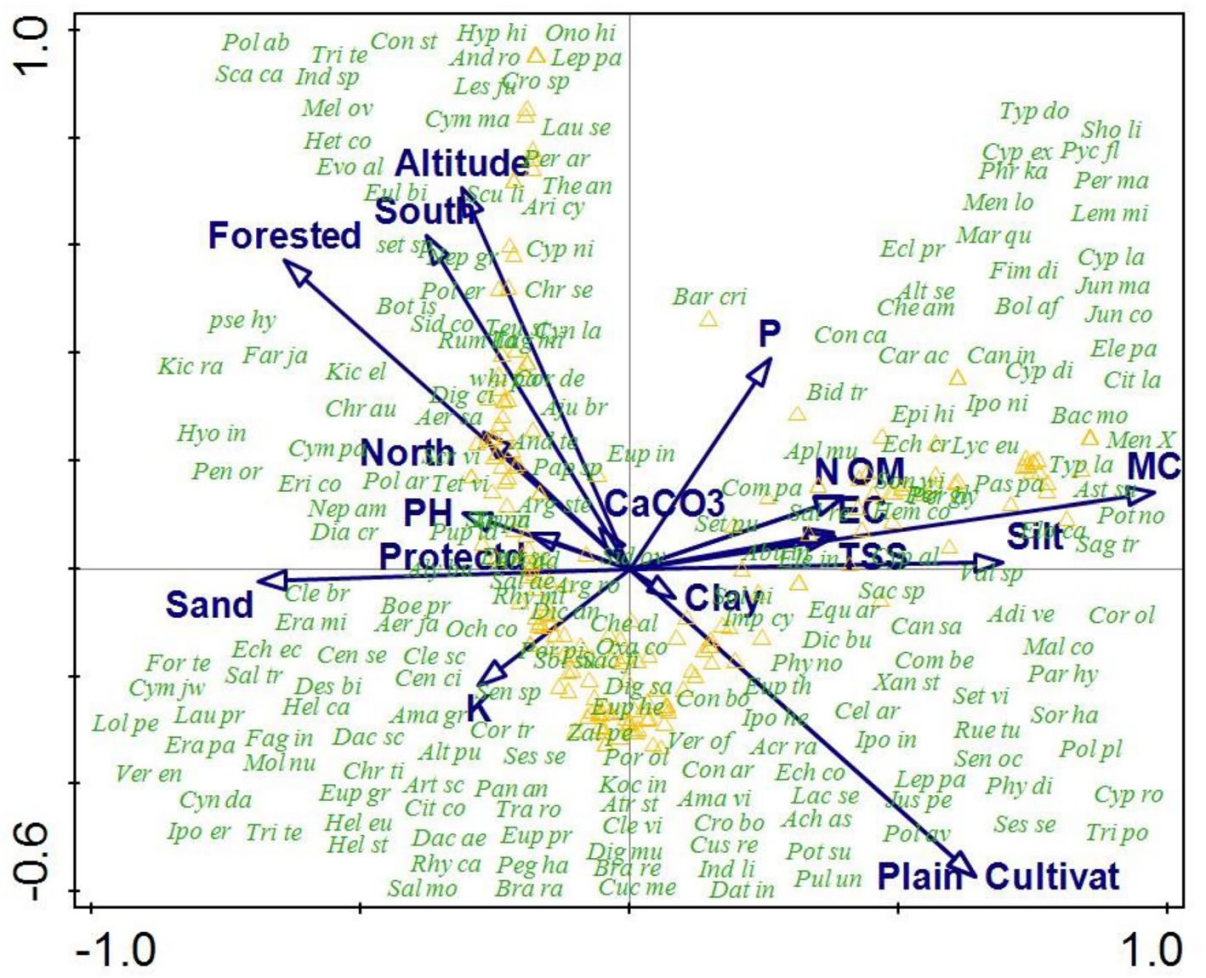
CCA biplot showing the distribution of the herbaceous plant species along the environmental factors.

To find out the influences of environmental variables on the species distribution, Canonical Correspondence Analysis (CCA) were used. Statistically, CCA analysis showed significant relation (*p* = 0.002) between environmental variables and species distribution. The first quadrant CCA (Biplot) revealed that higher number of species were clustered around the influence of available Moisture content (MC), higher concentration of silt, Total soluble salts (TSS), organic matter (OM), Nitrogen (N), Electrical conductivity and phosphorus. The species present in second quadrant of CCA biplot were clustered under the influences of Altitude, Aspect, pH and also by the type of habitat i.e. forested and protected habitat. The third quadrant of CCA biplot showed that these species were influenced by higher concentration of sand and potassium (K). The fourth quadrant of CCA biplot showed that these species were grouped in the cultivated plains of the area. The plain topography and the cultivation practices supports the growth of species present in quadrant 4 (Table 4) (Fig. 7).

**Table 4:** Summary of the CCA analysis.

Axes	1	2	3	4	Total inertia
Eigen value	0.779	0.606	0.462	0.283	7.2
Species-environment correlations	0.990	0.968	0.940	0.938	
Cumulative percentage variance of species data	10.72	19.5	25.40	29.30	
Cumulative percentage explained fitted variation	19.92	35.40	47.2	54.4	
F-ratio	1.5				
*P* value	0.002

#### CCA analysis of the major group and environmental factors

**Figure 8:**
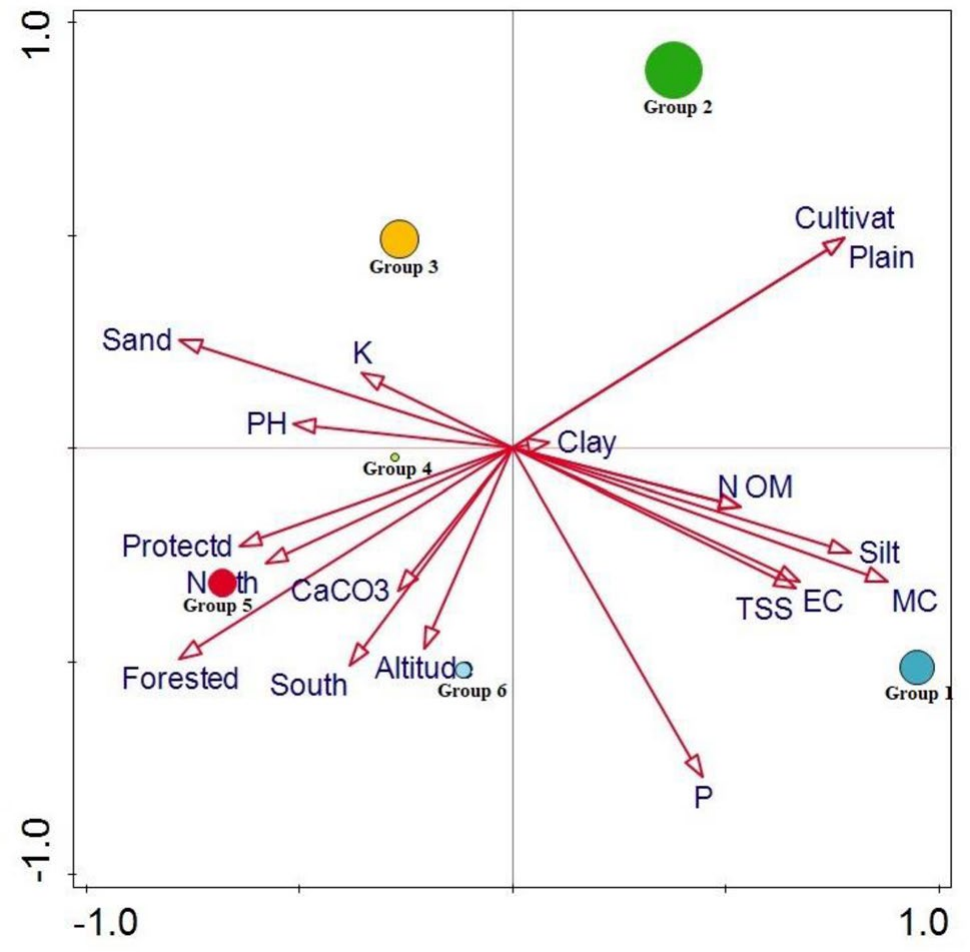
CCA plot showing the impact of different ecological factors on the yielded six major plant groups of the area. Number of species of a group were shown by the size of the circle.

Our cluster analysis yielded 6 different groups based on floristic similarity. The CCA graph showed that group 2 was more related to the plain topography and cultivated status of the habitats. the high concentration of K, pH, and sand showed their influences on group 3, CaCO_3,_ Aspect, Forested nature and Altitude showed its impact on the group 4, group 5 and group 6. While high soil moisture contents, silt and Phosphorus control the distribution of group 1 (Fig. 8).

## Discussion

The aggregation of different plant species into various major groups is basically response to the existing environmental conditions Haq et al.^13^. The present sub-tropical region owes a variety of different ecological habitats. from plains to hills, from xeric to moist, from low to high altitude and from cultivated to forested areas, because of the availability of a variety of habitats the area supports the growth of many distinct plant groups. In the present study a total of 216 summer herbaceous plant species were recorded from 40 different selected ecological habitats. vegetation study of an area includes the classification, distribution and the relationships of the existing plant cover with the prevailing ecological condition Iqbal et al.^14^. The microclimatic conditions of the district vary from the low altitude plains with relatively high soil moisture contents up to the high-altitude hilly topography. The collected vegetation data from such highly diversified area (40 sites) were classified through multivariate statistical analysis via PC-ORD v.5 software. The whole data of the 40 sites and 216 herbaceous species were sorted in six major plant groups on the basis of there floristic similarities. These six major plants groups were named after the dominant species on the basis of IV in each group. The six different major plant groups of the area were Group 1. *Paspalum paspalodes, Alternanthera sessilis, Typha domingensis,* high soil moisture content and high silt concentration of these sites influence this plant group. Group 2. *Cynodon dactylon, Parthenium hysterophorus, Brachiaria ramosa,* this plant group was prevailing due to the cultivated status and plain topography of the sites with moderate soil moisture content. Group 3. *Cynodon dactylon, Eragrostis minor, Cymbopogon jwarancusa,* the species of this plant groups clustered under the influence of high sand concentration which in term holds low soil moisture contents. Group 4. *Cymbopogon jwarancusa, Aristida adscensionis*, *Boerhavia procumbens,* this plant group was found in the rangelands which have relatively high altitude. group 5. *Cymbopogon jwarancusa, Aristida adscensionis, Pennisetum orientale,*this plant group was established in the low altitude hills of the area. Group 6. *Heteropogon contortus*, *Bothrriochloa ischaemum*, *Chrysopogon serrulatus*. The plant life of the species of this group were influenced by the high altitude. These groups were formed due to there floristic similarities and were named after the dominant species of the groups. These major vegetation groups types exist in the area due to the specific site conditions (Environmental factors) Rahman et al.^15^. The grouping of plants species into identifiable plant groups is the consequences of the prevailing environmental conditions Giuponi et al.^16^.

In the sub-tropical regions, ecological studies, in particular vegetation analysis are important for the understanding of underlying relationship between the plant species and environmental factors Lolila et al.^17^. Mainly the environmental factors of an area included status, soil characteristics, topography and altitude Waheed et al.^18^. CANOCO version 5.1 was used to analyze the effect of different prevailing ecological conditions on the distribution of the plant species in the area. To find out the impact of ecological factors on the existing plants, the DCA (indirect gradient) and CCA (direct gradient) analysis were often used in many vegetational studies such as Ali et al.^19^.

The DCA graphs are interpretable in the multivariate analysis of the vegetation. It shows the distribution of the plant species in the research area Kobal et al.^20^. The present DCA diagrams of the stands and species showed their distribution in relation to topography, altitude, soil moisture content and status whether it was cultivated or forested. The present DCA graphs beautifully arranged the whole data into highly interpretable patterns. The species and stands which were located at the left sites of the graphs were having relatively high soil moisture contents with plain topography and low altitude. While the species and stands which were located at the right side of the DCA graph were having low moisture content with hilly topography with relatively high altitude.

In the present study the CCA analysis showed that soil moisture content, altitude, topography, status and the edaphic factors were the strong influencing factors which were acting as the driving environmental factors which checked the distribution of different plants species in different ecological habitats Majeed et al.^21^. The anthropogenic factors also played key role in the structuring of vegetation at different habitats Jamil et al.^22^. The present study also showed that the plain areas with high moisture contents and high silt particles supports the growth of more diverse plants groups as compared to the xeric areas with hilly nature and high sand particles Ali et al.^23^. Some of the species were only restricted to the high altitude’s habitats Mumshad et al.^4^. Plant species by origins restricted to specific environmental condition and be present in the particular habitats due to the presence of certain principal ecological components due to which a specific plant group is formed Rahman et al.^15^. The present CCA analysis showed the influences of different environmental factors on the clustering of different plants species into identifiable plant groups. The CCA diagram showed that the six major plants groups were strongly influenced by the existing biotic and abiotic conditions due to which these plants groups were formed. Our statement about the role of biotic and abiotic conditions in the aggregation of different plant species into specific group were supported by many other researches such as Hamdy et al.^24^; Wali et al.^25^; Zeb et al.^3^; Hussain et al.^26^ and Anwar et al.^27^.

DCA and CCA analysis are widely used ordination techniques in vegetation ecology and are frequently used for accurate determination of the distinct unit and significance of the relationship between the plant species and environmental factors. The present analysis revealed that, among environmental factors MC, topography, status (Forested or cultivated), altitude and soil texture has great influences on the distribution of plant species Mansoor et al.^28^, Khan et al.^29^. CCA analysis was employed to know the vegetation-environment inter-relationships Iqbal et al.^14^; Khan et al.^30^ and Ilyas et al.^31^. It provides a biplot that directly showed the influences of different environmental factors on the distribution of plant species Mehmood et al.^32^; Khan et al.^33^; Shaheen et al.^34^ and Zareen et al.^35^.

## Conclusion

In the present study a total of 216 herbaceous species were recorded from forty different sites during summer season. Six major groups were recognized that were associated with particular environmental factors. Environmental factors include biotic, edaphic and topographic factors. DCA results further clarified the six major plant groups. CCA analysis confirmed the significance of the environmental factors which moulds the vegetation structure and distribution of the plant species in the area. The present study explored the vegetation pattern and vegetation-environment interrelationship which will be helpful to plan suitable measures for the conservation of the vegetation structure (Supplementary file 1).

### Supplementary Information

Supplementary Information.

## Data Availability

All data generated or analysed during this study are included in this published article.
