# Supplementary material for: Multivariate analysis of the summer herbaceous vegetation and environmental factors of the sub-tropical region
Source: Sci Rep. 2024 Jul 8;14:15657. doi: 10.1038/s41598-024-63780-8 (PMC11231133; doi:10.1038/s41598-024-63780-8)
Supplement: Supplementary file 1 — Supplementary Information. [file 41598_2024_63780_MOESM1_ESM.docx]

**Ecological evaluation of the vegetation structure and distribution of the summer herbaceous species at various habitats of ecotonal region**

Supplementary material

Table 1

| Site name | Clay | Silt | sand | Texture | pH | EC | TSS | CaCO3 | OM | N | P | K | MC |
| --- | --- | --- | --- | --- | --- | --- | --- | --- | --- | --- | --- | --- | --- |
| Perennial stream at Torsum | 11 | 72 | 17 | Loam | 8.3 | 0.14 | 0.044 | 8.25 | 2.2 | 0.11 | 2.6 | 130 | 45.5 |
| Perennial stream Toi | 11 | 54 | 35 | Siltloam | 7.9 | 0.08 | 0.025 | 8.75 | 3.08 | 0.154 | 4.7 | 120 | 37.3 |
| Perennial stream at Nasratkhel | 11 | 76 | 13 | Loam | 8.2 | 0.22 | 0.07 | 8.5 | 2.76 | 0.138 | 12.9 | 68 | 36.2 |
| Seasonal streams at Tanda | 11 | 46 | 43 | Loam | 8.25 | 0.12 | 0.038 | 6.5 | 2.41 | 0.12 | 7.1 | 240 | 26.2 |
| Seasonal streams at Doda | 11 | 44 | 45 | Siltloam | 8.3 | 0.18 | 0.058 | 8.75 | 2 | 0.1 | 3.7 | 122 | 25.5 |
| Tanda Dam banks | 11 | 52 | 37 | Loam | 8.4 | 0.11 | 0.035 | 8.75 | 2.76 | 0.138 | 3.9 | 124 | 15.4 |
| Wetlands | 11 | 71 | 16 | Loam | 8.3 | 0.14 | 0.044 | 9.25 | 2.2 | 0.11 | 10.2 | 129 | 47.2 |
| Irrigated lands of Tanda | 11 | 46 | 43 | Loam | 8.6 | 0.06 | 0.019 | 8.75 | 3.1 | 0.155 | 3.8 | 84 | 19.2 |
| Irrigated lands of Bana | 11 | 40 | 49 | Loam | 8.1 | 0.12 | 0.038 | 9 | 1.72 | 0.086 | 4.5 | 148 | 14.4 |
| Irrigated lands of Doda | 11 | 66 | 23 | Siltloam | 8.4 | 0.11 | 0.035 | 9.25 | 2.76 | 0.138 | 8 | 562 | 16.1 |
| Irrigated lands Gulhasan Banda | 11 | 28 | 61 | Sandyloam | 8.5 | 0.09 | 0.029 | 7.5 | 1.03 | 0.051 | 6.2 | 326 | 11.12 |
| Irrigated lands of Savo | 11 | 44 | 45 | Loam | 8.2 | 0.13 | 0.041 | 10 | 1.38 | 0.067 | 6.1 | 234 | 16.3 |
| Non-Irrigated lands of khushalgarh | 11 | 20 | 69 | Sandyloam | 8.7 | 0.05 | 0.016 | 7.75 | 0.34 | 0.017 | 7.4 | 120 | 9.2 |
| Non-Irrigated lands of Lokhari | 11 | 30 | 59 | Sandyloam | 8.3 | 0.07 | 0.022 | 6.5 | 1.65 | 0.082 | 4.5 | 210 | 9.9 |
| Non-Irrigated lands of Darmalak | 11 | 30 | 59 | Sandyloam | 8.3 | 0.14 | 0.044 | 9.25 | 1.38 | 0.069 | 4.7 | 298 | 10.5 |
| Non-Irrigated lands of Paka | 11 | 14 | 75 | Sandyloam | 8.4 | 0.08 | 0.025 | 9.25 | 0.69 | 0.034 | 2.4 | 92 | 8.5 |
| Non-Irrigated lands of Smari | 10 | 27 | 63 | Sandyloam | 8.3 | 0.17 | 0.05 | 8 | 2.41 | 0.12 | 5.5 | 164 | 9.8 |
| Non-Irrigated lands of Sudal | 11 | 24 | 65 | Sandyloam | 8.1 | 0.14 | 0.044 | 7.25 | 1.38 | 0.069 | 2.6 | 400 | 9.4 |
| Non-Irrigated lands of Tulang | 10 | 30 | 60 | Sandyloam | 8 | 0.23 | 0.073 | 9.25 | 1.72 | 0.086 | 3.4 | 212 | 8.6 |
| Non-Irrigated lands of Zyara | 11 | 24 | 65 | Sandyloam | 8.5 | 0.05 | 0.016 | 8 | 0.69 | 0.034 | 7.3 | 110 | 8.3 |
| Nonprotected rangelands at Ziaratsheikh | 11 | 26 | 63 | Sandyloam | 8.4 | 0.14 | 0.044 | 6.5 | 0.34 | 0.017 | 4 | 404 | 8.2 |
| Nonprotected rangelands at Krapa | 11 | 40 | 49 | Loam | 8.5 | 0.05 | 0.016 | 7.25 | 0.69 | 0.034 | 3.4 | 172 | 8 |
| Nonprotected rangelands at Merai Payan | 11 | 40 | 49 | Loam | 8 | 0.11 | 0.035 | 9.25 | 2.07 | 0.103 | 7.4 | 510 | 8.6 |
| Nonprotected rangelands Shadikhel | 11 | 58 | 31 | Siltloam | 8.1 | 0.16 | 0.051 | 8.75 | 2.07 | 0.103 | 3.7 | 392 | 8.1 |
| Protected rangelands of kotal Park | 11 | 36 | 53 | Sandyloam | 8 | 0.11 | 0.035 | 9 | 2.76 | 0.138 | 3.9 | 464 | 7.8 |
| Protected rangelands of Tanda dam Park | 11 | 44 | 45 | Loam | 8.6 | 0.05 | 0.016 | 8.75 | 1.72 | 0.086 | 5.5 | 152 | 8.9 |
| Protected rangelands of Togh Park | 11 | 26 | 63 | Sandyloam | 8.5 | 0.06 | 0.019 | 9.5 | 1.38 | 0.069 | 7.3 | 498 | 8.4 |
| South aspect of Merai high Hills | 11 | 22 | 67 | Sandyloam | 8.3 | 0.11 | 0.035 | 8.25 | 2.07 | 0.103 | 10.2 | 104 | 9.2 |
| South aspect of Badasum Hills | 11 | 22 | 67 | Sandyloam | 8.9 | 0.08 | 0.025 | 8.75 | 0.62 | 0.031 | 2.4 | 70 | 8.1 |
| South aspect of Kotal Hills | 11 | 36 | 53 | Sandyloam | 8.2 | 0.06 | 0.019 | 8.25 | 2.76 | 0.138 | 5.3 | 160 | 8.2 |
| South aspect of Merai low Hills | 11 | 40 | 49 | Loam | 8.1 | 0.14 | 0.044 | 9.75 | 0.48 | 0.024 | 6 | 198 | 8.3 |
| South aspect of Shakardara Hills | 11 | 30 | 59 | Sandyloam | 8.5 | 0.06 | 0.019 | 9.75 | 2.07 | 0.103 | 4.5 | 100 | 7.9 |
| South aspect of Smari Bala Hills | 10 | 19 | 71 | Sandyloam | 8.9 | 0.19 | 0.061 | 8.75 | 0.55 | 0.027 | 4.6 | 156 | 9.3 |
| South aspect of Tulang Hills | 11 | 22 | 67 | Sandyloam | 8.3 | 0.08 | 0.025 | 6.75 | 2.41 | 0.12 | 5.5 | 192 | 7.8 |
| South aspect of Zameerdam | 11 | 52 | 37 | Siltloam | 8.5 | 0.05 | 0.016 | 9.25 | 1.38 | 0.069 | 8.1 | 522 | 8.2 |
| North aspect of Ghorzai Hills | 11 | 34 | 55 | Sandyloam | 8.3 | 0.11 | 0.035 | 7.5 | 0.69 | 0.034 | 5.2 | 166 | 9.5 |
| North aspect of Tanda Hills | 11 | 42 | 47 | Loam | 8.4 | 0.07 | 0.022 | 9.25 | 3.1 | 0.155 | 4.5 | 158 | 9.6 |
| North aspect of Shakardara Hills | 11 | 30 | 49 | Sandyloam | 8.5 | 0.09 | 0.029 | 9.5 | 1.72 | 0.086 | 6 | 456 | 9.9 |
| North aspect of Tulang Hills | 11 | 22 | 67 | Sandyloam | 8.5 | 0.05 | 0.016 | 9.25 | 2.07 | 0.103 | 8.2 | 134 | 8.2 |
| North aspect of Zameerdam Hills | 11 | 40 | 49 | Loam | 8.7 | 0.07 | 0.022 | 9.5 | 3.1 | 0.155 | 4.5 | 436 | 8.9 |

Table 2

| Species name | Family | LF | LS | S1 | S2 | S3 | S4 | S5 | S6 | S7 | S8 | S9 | S10 | S11 | S12 | S13 | S14 | S15 | S16 | S17 | S18 | S19 | S20 | S21 | S22 | S23 | S24 | S25 | S26 | S27 | S28 | S29 | S30 | S31 | S32 | S33 | S34 | S35 | S36 | S37 | S38 | S39 | S40 | TIV |
| --- | --- | --- | --- | --- | --- | --- | --- | --- | --- | --- | --- | --- | --- | --- | --- | --- | --- | --- | --- | --- | --- | --- | --- | --- | --- | --- | --- | --- | --- | --- | --- | --- | --- | --- | --- | --- | --- | --- | --- | --- | --- | --- | --- | --- |
| *Abutilon indicum* (L.) Sweet. | Malvaceae | H | Mic | 3.1 |  |  |  |  |  |  |  |  |  |  |  |  |  |  |  |  |  |  |  |  |  |  |  |  |  | 7.7 |  |  |  |  |  |  |  |  |  |  |  |  |  | 10.8 |
| *Achyranthus aspera* L. | Amaranthaceae | Th | Mic |  |  |  | 3.1 | 7.8 |  |  | 8.1 | 11.1 | 25.1 |  | 14.9 |  | 6.7 | 3.7 | 9.2 | 4.7 | 3.4 |  |  |  | 1.8 |  |  |  |  |  |  |  |  |  |  |  |  |  |  |  |  |  |  | 99.6 |
| *Acrachne racemosa* (Heyne ex Roem. & Schult.) Ohwi. | Poaceae | Th | Mic |  |  |  |  |  |  |  | 5.3 | 2.4 |  |  |  |  |  |  |  |  |  |  |  |  |  |  |  |  |  |  |  |  |  |  |  |  |  |  |  |  |  |  |  | 7.6 |
| *Adiantum capillus-veneris* L. | Adiantaceae | H | N |  |  |  |  | 3.7 |  |  |  |  |  |  |  |  |  |  |  |  |  |  |  |  |  |  |  |  |  |  |  |  |  |  |  |  |  |  |  |  |  |  |  | 3.7 |
| *Aerva javanica* (Burm. f.) Schult. | Amaranthaceae | Ch | Mic |  |  |  |  |  |  |  |  |  |  |  |  | 5.4 | 11.7 |  | 12.7 |  |  |  |  |  | 9.2 |  |  |  |  |  |  | 10.7 |  |  | 6.7 |  |  | 9.7 |  |  |  |  |  | 66.1 |
| *Aerva sanguinolenta* (L.) Blume. | Amaranthaceae | Ch | Mic |  |  |  |  |  |  |  |  |  |  |  |  |  |  |  |  |  |  |  |  |  |  |  |  |  |  |  |  |  |  |  | 9.5 |  |  |  | 11.3 |  |  |  |  | 20.8 |
| *Ajuga bracteosa* Wall. ex Benth. | Lamiaceae | H | N |  |  |  |  |  |  |  |  |  |  |  |  |  |  |  |  |  |  |  |  |  |  | 8.0 |  |  |  |  |  |  |  | 7.2 |  |  |  |  |  |  |  |  |  | 15.2 |
| *Alternanthera pungens* Kunth. | Amaranthaceae | Th | N |  |  |  |  |  |  |  |  | 9.4 | 5.3 |  | 6.8 |  |  | 3.4 | 5.7 | 3.9 | 3.3 |  |  |  |  |  |  |  |  |  |  |  |  |  |  |  |  |  |  |  |  |  |  | 37.9 |
| *Alternanthera sessilis* (L.) R. Br. ex DC. | Amaranthaceae | Th | N | 16.1 | 33.8 | 20.5 | 24.8 | 22.7 |  | 3.5 |  |  |  |  |  |  |  |  |  |  |  |  |  |  |  |  |  |  |  |  |  |  |  |  |  |  |  |  |  |  |  |  |  | 121.3 |
| *Alysicarpus bupleurifolius* (L.) DC. | Papilionaceae | H | N |  |  |  |  |  |  |  |  |  |  |  |  |  |  |  |  |  |  |  |  |  |  |  |  |  |  |  |  |  |  |  |  |  |  | 6.1 |  |  |  |  |  | 6.1 |
| *Amaranthus graecizans* L. | Amaranthaceae | Th | N |  |  |  |  |  |  |  |  | 10.1 | 1.8 |  | 4.6 |  |  | 3.4 | 4.4 |  | 1.7 |  | 8.8 | 4.0 | 2.0 |  |  |  |  |  |  |  |  |  |  |  |  |  |  |  |  |  |  | 40.8 |
| *Amaranthus viridis* L. | Amaranthaceae | Th | Mic |  |  |  |  |  |  |  |  | 3.9 | 3.4 |  | 2.9 |  |  | 3.7 |  |  | 1.7 |  |  |  |  |  |  |  |  |  |  |  |  |  |  |  |  |  |  |  |  |  |  | 15.6 |
| *Andrachne telephiodes* L. | Euphorbiaceae | H | N |  |  |  |  |  |  |  |  |  |  |  |  |  |  |  |  |  |  |  |  |  |  |  |  |  |  |  |  |  |  |  |  |  |  |  | 6.9 | 5.5 |  |  |  | 12.3 |
| *Androsace rotundifolia* Hardwicke. | Primulaceae | H | N |  |  |  |  |  |  |  |  |  |  |  |  |  |  |  |  |  |  |  |  |  |  |  |  |  |  |  | 7.7 |  |  |  |  |  |  |  |  |  |  |  |  | 7.7 |
| *Apluda mutica* L. | Poaceae | H | Mic |  |  | 34.7 | 14.4 | 14.6 |  |  |  |  |  |  |  |  |  |  |  |  |  |  |  |  |  |  |  |  |  |  |  |  |  |  |  |  |  |  |  |  |  |  |  | 63.6 |
| *Argyrolobium roseum* (Camb.) Jaub. | Papilionaceae | H | N |  |  |  |  |  | 3.8 |  |  |  |  |  |  |  |  |  |  |  |  |  |  |  |  |  |  |  |  |  |  | 2.3 |  |  |  |  |  |  |  |  |  |  | 5.5 | 11.6 |
| *Argyrolobium stenophyllum* Boiss. | Papilionaceae | H | N |  |  |  |  |  |  |  |  |  |  |  |  |  |  |  |  |  |  |  |  |  |  | 2.3 | 9.7 |  |  |  |  | 2.5 |  |  | 2.9 | 2.4 | 3.6 |  |  |  |  |  |  | 23.4 |
| *Aristida adscensionis* L. | Poaceae | Th | Mic |  |  |  |  |  |  |  |  |  |  |  |  | 4.2 | 8.0 | 6.9 | 13.6 | 21.9 | 4.8 | 4.9 | 4.2 | 29.5 | 5.4 | 6.3 | 4.8 | 56.3 | 70.6 | 50.5 |  | 16.8 | 39.5 | 20.5 | 27.9 | 2.2 | 35.1 | 32.9 | 17.8 | 24.2 | 30.0 | 26.1 | 28.9 | 593.6 |
| *Aristida cyanantha* Nees. ex Steud. | Poaceae | Ch | Mes |  |  |  |  |  |  |  |  |  |  |  |  |  |  |  |  |  |  |  |  |  |  |  |  |  |  |  |  |  |  |  |  | 32.6 |  |  |  |  |  |  |  | 32.6 |
| *Artemisia scoparia* Waldst. & Kit. | Asteraceae | Ch | N |  |  |  |  |  |  |  |  |  |  |  |  |  |  |  |  |  | 10.2 |  |  |  |  |  |  |  |  |  |  |  |  |  |  |  |  |  |  |  |  |  |  | 10.2 |
| *Leptorhabdos parviflora* (Benth.) Benth. | Scrophulariaceae | Th | Mic |  |  |  |  |  |  |  |  |  |  |  |  |  |  |  |  |  |  |  |  |  |  |  |  |  |  |  | 3.7 |  |  |  |  |  |  |  |  |  |  |  |  | 3.7 |
| *Aster subulatus* Michaux. | Asteraceae | Th | Mic | 11.5 | 9.7 | 17.3 | 9.6 | 17.8 |  | 2.3 | 3.3 |  | 2.9 |  |  |  |  |  |  |  |  |  |  |  |  |  |  |  |  |  |  |  |  |  |  |  |  |  |  |  |  |  |  | 74.5 |
| *Atriplex stocksii* Boiss. | Chenopodiaceae | Th | Mic |  |  |  |  |  |  |  |  | 3.4 | 6.1 |  | 2.9 |  |  | 11.1 |  |  | 17.3 |  |  |  |  |  |  |  |  |  |  |  |  |  |  |  |  |  |  |  |  |  |  | 40.8 |
| *Bacopa monnieri* (L.) Pennell. | Plantaginaceae | Th | N |  |  |  |  |  |  | 17.7 |  |  |  |  |  |  |  |  |  |  |  |  |  |  |  |  |  |  |  |  |  |  |  |  |  |  |  |  |  |  |  |  |  | 17.7 |
| *Barleria cristata* L. | Acanthaceae | H | Mic |  |  | 12.8 | 12.0 |  |  |  |  |  |  |  |  |  |  |  |  |  |  |  |  |  |  |  |  |  |  |  | 3.9 |  |  | 4.8 |  | 4.8 |  |  |  |  |  |  |  | 38.2 |
| *Bidens tripartita* L. | Asteraceae | Th | Mic |  |  | 12.8 | 12.0 |  |  |  |  |  |  |  |  |  |  |  |  |  |  |  |  |  |  |  |  |  |  |  |  |  |  | 4.8 |  |  |  |  |  |  |  |  |  | 29.5 |
| *Boerhavia procumbens* Banks. ex Roxb. | Nyctaginaceae | H | N |  |  |  |  |  |  |  |  | 4.6 | 3.5 |  | 6.2 | 4.4 | 9.3 | 9.1 | 37.5 |  | 3.0 | 7.1 | 4.7 | 16.3 | 9.5 | 21.6 | 14.8 | 10.2 | 21.1 | 14.9 |  | 14.4 |  |  | 7.7 |  | 12.9 |  | 7.9 |  |  |  |  | 240.7 |
| *Bolboschoenus affinis* (Roth.) Droboy. | Cyperaceae | G | Mic |  | 15.6 |  |  |  |  | 58.6 |  |  |  |  |  |  |  |  |  |  |  |  |  |  |  |  |  |  |  |  |  |  |  |  |  |  |  |  |  |  |  |  |  | 74.2 |
| *Bothrriochloa ischaemum* (L.) Keng. | Poaceae | Ch | Mic |  |  |  |  |  |  |  |  |  |  |  |  |  |  |  |  |  |  |  |  |  |  |  |  |  |  |  |  | 6.2 |  | 16.2 |  | 52.5 |  |  |  |  |  |  | 24.4 | 99.3 |
| *Brachiaria ramosa* (L.) Stapf. | Poaceae | Th | Mic |  |  |  |  |  |  |  | 23.6 | 11.3 | 10.3 | 12.0 | 5.1 | 7.0 |  |  |  |  | 1.8 |  | 12.5 |  | 7.0 | 3.9 |  |  |  |  |  |  |  | 5.1 |  |  |  |  |  |  |  |  |  | 99.6 |
| *Brachiaria reptans* (L.) Gardner & Hubbard. | Poaceae | Th | N |  |  |  |  |  | 9.1 |  | 17.6 | 14.9 | 29.7 | 3.1 | 26.4 |  | 10.0 | 17.0 |  | 23.4 | 7.2 | 9.2 | 4.2 |  |  |  |  |  |  |  |  |  |  |  |  |  |  |  |  |  |  |  |  | 171.9 |
| *Canna indica* L. | Cannaceae | Ch | Meg |  |  | 21.1 |  |  |  |  |  |  |  |  |  |  |  |  |  |  |  |  |  |  |  |  |  |  |  |  |  |  |  |  |  |  |  |  |  |  |  |  |  | 21.1 |
| *Cannabis sativa* L. | Canabinaceae | Th | Mic | 16.0 |  |  |  | 4.7 |  |  |  |  | 4.9 |  |  |  |  |  |  |  |  |  |  |  |  |  |  |  |  |  |  |  |  |  |  |  |  |  |  |  |  |  |  | 25.6 |
| *Carex acutiformis* Ehrh. | Cyperaceae | Ch | Mic |  |  | 5.5 |  |  |  |  |  |  |  |  |  |  |  |  |  |  |  |  |  |  |  |  |  |  |  |  |  |  |  |  |  |  |  |  |  |  |  |  |  | 5.5 |
| *Celosia argentea* L. | Amaranthaceae | Th | Mic |  |  |  |  |  |  |  | 5.3 |  |  |  |  |  |  |  |  |  |  |  |  |  |  |  |  |  |  |  |  |  |  |  |  |  |  |  |  |  |  |  |  | 5.3 |
| *Cenchrus ciliaris* L. | Poaceae | Ch | Mic |  |  |  |  |  |  |  |  |  |  | 7.8 | 10.9 | 8.8 | 6.9 | 5.2 | 21.0 |  | 6.5 | 14.3 | 14.4 | 14.3 | 9.0 |  | 21.7 |  |  | 7.6 |  |  |  | 2.4 |  |  |  |  | 3.9 | 8.9 |  |  |  | 163.7 |
| *Cenchrus setigerus* Vahl. | Poaceae | Th | Mic |  |  |  |  |  |  |  |  |  |  |  |  |  |  |  |  |  |  |  |  |  | 11.8 | 8.8 |  |  |  |  |  |  |  |  |  |  |  |  |  |  |  |  |  | 20.6 |
| *Chenopodium album* L. | Chenopodiaceae | Th | Mic |  |  |  |  |  |  |  |  |  | 3.2 |  | 3.5 |  |  |  |  |  |  |  |  |  |  |  |  | 24.6 |  |  |  |  |  |  |  |  |  |  |  |  |  |  |  | 31.4 |
| *Chenopodium ambrosoides* L. | Chenopodiaceae | Ch | Mic | 6.9 | 4.0 | 2.9 | 7.8 | 3.1 |  |  |  |  |  |  |  |  |  |  |  |  |  |  |  |  |  |  |  |  |  |  |  |  |  |  |  |  |  |  |  |  |  |  |  | 24.8 |
| *Chrozophora tinctoria* (L.) Raffin. | Euphorbiaceae | Th | Mic |  |  |  |  |  |  |  |  |  |  |  | 5.9 | 9.3 | 3.5 | 1.9 |  |  | 6.3 | 9.6 | 11.0 |  | 10.1 |  |  |  |  |  |  |  |  |  |  |  |  |  |  |  |  |  |  | 57.6 |
| *Chrysopogon aucheri* (Boiss) Stapf. | Poaceae | Ch | Mic |  |  |  |  |  |  |  |  |  |  |  |  |  |  |  |  |  |  |  |  |  |  |  |  |  | 6.3 | 68.8 |  | 8.4 | 29.8 | 9.1 | 14.4 | 43.6 | 27.2 | 26.7 | 9.2 |  | 7.1 | 30.2 | 20.4 | 301.2 |
| *Chrysopogon serrulatus* Trin. | Poaceae | Ch | Mic |  |  |  |  |  |  |  |  |  |  |  |  |  |  |  |  |  |  |  |  |  |  |  |  |  |  |  | 54.1 |  |  | 5.5 |  |  | 12.9 |  |  |  |  |  | 22.0 | 94.5 |
| *Citrullus colocynthis* (L.) Schard. | Cucurbitaceae | Th | Mic |  |  |  |  |  |  |  |  |  |  | 5.1 | 2.9 |  | 2.0 |  |  |  | 3.0 | 4.3 | 6.9 |  |  |  |  |  |  |  |  |  |  |  |  |  |  |  |  |  |  |  |  | 24.2 |
| *Citrullus lanatus* (Thunb.) Mats. & Nakai. | Cucurbitaceae | Th | Mic |  |  |  |  |  |  | 3.4 |  |  |  |  |  |  |  |  |  |  |  |  |  |  |  |  |  |  |  |  |  |  |  |  |  |  |  |  |  |  |  |  |  | 3.4 |
| *Cleome brchycarpa* Vahl. ex DC. | Capparidaceae | Th | N |  |  |  |  |  |  |  |  |  |  |  |  |  |  |  |  |  |  |  |  |  |  |  | 4.8 |  |  |  |  |  |  |  | 9.5 |  |  |  |  |  |  |  |  | 14.3 |
| *Cleome scaposa* DC. | Capparidaceae | Th | N |  |  |  |  |  |  |  |  |  |  |  |  |  |  |  |  |  |  |  |  | 20.0 | 6.7 |  |  |  |  |  |  |  |  |  |  |  |  |  |  |  |  |  |  | 26.8 |
| *Cleome viscosa* L. | Capparidaceae | Th | Mic |  |  |  |  |  | 13.8 |  |  |  |  |  | 3.2 |  |  |  | 6.5 |  |  |  |  |  |  |  |  |  |  |  |  |  |  |  |  |  |  |  |  |  |  |  |  | 23.5 |
| *Commelina benghalensis* L. | Commelinaceae | G | Mic |  |  |  |  |  |  |  | 12.3 |  |  |  |  |  |  |  |  |  |  |  |  |  |  |  |  |  |  |  |  |  |  |  |  |  |  |  |  |  |  |  |  | 12.3 |
| *Commelina paludosa* Blume. | Commelinaceae | H | Mic |  |  |  | 4.4 |  |  |  |  |  |  |  |  |  |  |  |  |  |  |  |  |  |  |  |  |  |  |  |  |  |  |  |  |  |  |  |  |  |  |  |  | 4.4 |
| *Convolvulus arvensis* L. | Convolvulaceae | Th | Mic |  |  |  |  |  |  |  |  | 4.3 | 1.6 |  | 1.7 |  | 3.5 |  |  |  |  | 7.5 |  |  |  |  |  |  |  |  |  |  |  |  |  |  |  |  |  |  |  |  |  | 18.6 |
| *Conyza bonariensis* (L.) Cronquist. | Asteraceae | Ch | Mic |  |  |  |  |  |  |  | 3.3 | 3.6 | 1.6 | 2.8 | 1.8 |  |  |  |  | 4.8 | 1.7 |  |  |  |  |  |  |  |  |  |  |  |  |  |  |  |  |  |  |  | 4.6 |  |  | 24.3 |
| *Conyza canadensis* (L.) Cronquist. | Asteraceae | Th | Mes |  | 4.4 | 2.9 | 6.8 | 6.7 |  |  |  |  |  |  |  |  |  |  |  |  |  |  |  |  |  |  |  |  |  |  |  |  |  |  |  |  |  |  |  |  |  |  |  | 20.8 |
| *Conyza stricta* Willd. | Asteraceae | Th | N |  |  |  |  |  |  |  |  |  |  |  |  |  |  |  |  |  |  |  |  |  |  |  |  |  |  |  | 7.7 |  |  |  |  |  |  |  |  |  |  |  |  | 7.7 |
| *Corbichonia decumbens* (Forssk.) Exell. | Aizoaceae | Ch | N |  |  |  |  |  |  |  |  |  |  |  |  |  |  |  |  |  |  |  |  |  |  |  |  |  |  |  |  |  | 6.6 |  |  |  |  |  |  |  |  |  |  | 6.6 |
| *Corchorus olitorius* L. | Tiliaceae | Th | Mic |  |  |  |  |  | 6.1 |  | 5.1 |  | 7.0 |  |  |  |  |  |  |  |  |  |  |  | 1.6 |  |  |  |  |  |  |  |  |  |  |  |  | 5.3 |  |  |  |  |  | 25.2 |
| *Corchorus tridens* L. | Tiliaceae | Th | N |  |  |  |  |  | 30.3 |  |  |  |  |  |  | 7.0 | 7.4 |  | 8.3 |  | 1.7 | 4.7 |  | 3.6 | 8.8 | 5.0 | 4.8 |  |  |  |  |  |  |  | 9.6 |  |  |  |  |  |  |  |  | 91.1 |
| *Crotolaria sp.* | Papilionaceae | H | N |  |  |  |  |  |  |  |  |  |  |  |  |  |  |  |  |  |  |  |  |  |  |  |  |  |  |  | 5.9 |  |  |  |  |  |  |  |  |  |  |  |  | 5.9 |
| *Croton bonplandianus* Baill. | Euphorbiaceae | Th | Mic |  |  |  |  |  | 10.6 |  |  |  |  |  |  |  |  |  |  |  |  |  |  |  |  |  |  |  |  |  |  |  |  |  |  |  |  |  |  |  |  |  |  | 10.6 |
| *Cucumis melo* L. subsp. *Agrestis* (Naudin) Pangalo. | Cucurbitaceae | Th | Mic |  |  |  |  |  |  |  |  |  | 12.8 | 7.6 | 1.7 |  | 1.9 | 3.1 | 3.7 |  |  |  |  |  |  |  |  |  |  |  |  |  |  |  |  |  |  |  |  |  |  |  |  | 30.7 |
| *Cuscuta reflexa* Roxb. | Cuscutaceae | P | Aph |  |  |  |  |  | 7.9 |  |  |  |  |  |  |  |  |  |  |  |  |  |  |  |  |  |  |  |  |  |  |  |  |  |  |  |  |  |  |  |  |  |  | 7.9 |
| *Cymbopogon jwarancusa* (Jones) Schult. | Poaceae | Ch | Mic |  |  |  |  |  |  |  |  |  |  | 17.6 | 6.5 | 12.0 | 34.5 | 4.7 | 5.7 |  | 23.8 | 16.7 |  | 115.0 | 36.2 | 62.4 | 68.5 | 172.0 | 74.2 | 29.6 |  | 16.2 | 11.9 |  | 54.8 | 4.0 | 49.1 | 96.5 | 61.7 | 82.0 | 29.1 | 75.3 | 68.2 | 1228.4 |
| *Cymbopogon martini* (Roxb.) Wats. | Poaceae | Ch | Mic |  |  |  |  |  |  |  |  |  |  |  |  |  |  |  |  |  |  |  |  |  |  |  |  |  |  |  | 24.9 |  |  |  |  | 20.4 |  |  |  |  |  |  |  | 45.3 |
| *Cymbopogon* *pospischilii* (Schum.) Hubbard. | Poaceae | Ch | Mic |  |  |  |  |  |  |  |  |  |  |  |  |  |  |  |  |  |  |  |  |  |  |  |  |  |  |  |  | 4.7 |  |  |  |  |  |  |  |  |  |  |  | 4.7 |
| *Cynodon dactylon* (L.) Pers. | Poaceae | H | N |  | 8.1 | 6.6 | 7.9 | 16.8 | 11.1 |  | 24.1 | 44.0 | 22.4 | 47.8 | 39.0 | 51.4 | 35.4 | 41.2 | 18.2 | 45.9 | 29.9 | 51.5 | 53.1 | 11.2 | 21.3 | 3.8 | 15.2 | 17.0 | 6.3 | 3.4 |  | 7.0 |  | 7.2 |  |  |  |  | 6.9 |  | 5.4 |  |  | 659.1 |
| *Cynoglossum lanceolatum* Forssk. | Boraginaceae | H | Mic |  |  |  |  |  |  |  |  |  |  |  |  |  |  |  |  |  |  |  |  |  |  | 6.4 |  |  |  |  |  |  |  |  |  | 4.5 |  |  | 2.3 |  |  |  |  | 13.1 |
| *Cyperus alopecuroides* Rottb. | Cyperaceae | G | Mic | 1.9 |  |  | 7.4 | 6.2 |  |  |  |  |  |  |  |  |  |  |  |  |  |  |  |  |  |  |  |  |  |  |  |  |  |  |  |  |  |  |  |  |  |  |  | 15.6 |
| *Cyperus difformis* L. | Cyperaceae | G | Mic |  | 3.9 |  |  |  |  | 3.9 |  |  |  |  |  |  |  |  |  |  |  |  |  |  |  |  |  |  |  |  |  |  |  |  |  |  |  |  |  |  |  |  |  | 7.8 |
| *Cyperus exaltatus* Retz. | Cyperaceae | G | Mic |  | 8.4 |  |  |  |  | 6.6 |  |  |  |  |  |  |  |  |  |  |  |  |  |  |  |  |  |  |  |  |  |  |  |  |  |  |  |  |  |  |  |  |  | 15.1 |
| *Cyperus laevigatus* L. | Cyperaceae | G | N |  |  |  |  |  |  | 4.5 |  |  |  |  |  |  |  |  |  |  |  |  |  |  |  |  |  |  |  |  |  |  |  |  |  |  |  |  |  |  |  |  |  | 4.5 |
| *Cyperus niveus* Retz. | Cyperaceae | G | N |  |  |  |  |  |  |  |  |  |  |  |  |  |  |  |  |  |  |  |  |  |  |  |  |  |  |  | 23.9 | 2.5 |  | 4.8 |  | 15.2 |  |  |  |  |  | 12.5 |  | 59.0 |
| *Cyperus rotundus* L. | Cyperaceae | G | Mic |  | 2.8 | 4.7 | 3.7 |  | 16.3 | 4.1 | 7.6 | 4.5 | 1.6 | 29.7 | 22.1 | 25.9 | 16.9 | 25.9 | 10.9 | 23.4 | 11.1 | 23.7 | 21.3 | 7.2 | 7.4 |  |  |  |  |  |  |  |  | 7.4 |  |  |  |  |  |  |  |  |  | 278.2 |
| *Dactyloctenium aegyptium* (L.) Willd. | Poaceae | Th | N |  |  |  |  |  |  |  |  |  | 7.5 | 13.4 | 3.8 | 8.5 | 6.9 | 5.4 |  |  | 6.3 |  |  |  | 6.3 |  |  |  |  |  |  |  |  |  |  |  |  |  |  |  |  |  |  | 58.2 |
| *Dactyloctenium scindicum* Boiss. | Poaceae | H | N |  |  |  |  |  |  |  |  |  |  |  |  | 4.4 | 3.9 | 3.1 |  |  | 3.3 |  |  |  | 22.0 |  | 21.8 |  |  | 6.9 |  | 44.8 |  |  | 2.6 |  |  |  |  |  |  |  |  | 112.7 |
| *Datura innoxia* Miller. | Solanaceae | Th | Mes |  |  |  |  | 2.7 | 9.6 |  |  | 12.1 | 9.5 |  | 3.8 | 4.2 |  | 1.9 |  |  |  |  |  |  | 3.2 |  |  |  |  |  |  |  |  |  |  |  |  |  |  |  |  |  |  | 46.9 |
| *Papilionaceae* | Papilionaceae | Ch | Mic |  |  |  |  |  |  |  |  |  |  |  |  |  |  |  |  |  |  |  |  |  |  |  |  |  |  |  |  |  |  |  |  |  |  |  |  |  | 4.6 |  |  | 4.6 |
| *Desmostachya bippinata* (L.) Stapf. | Poaceae | Ch | Mic |  |  |  |  |  |  |  |  | 9.6 | 7.6 | 19.8 |  | 4.9 | 3.9 |  |  |  | 28.4 |  |  |  |  |  |  |  |  |  |  | 9.6 |  |  | 4.4 |  |  |  |  |  |  |  |  | 88.2 |
| *Dianthus crinitus* Sm. | Caryophyllaceae | H | Mic |  |  |  |  |  |  |  |  |  |  |  |  |  |  |  |  |  |  |  |  |  |  | 6.5 |  |  |  |  |  |  |  |  |  |  |  |  |  |  |  |  |  | 6.5 |
| *Dichanthium annulatum* (Forssk.) Stapf. | Poaceae | H | Mic |  |  |  |  | 3.1 |  |  | 8.4 |  | 3.2 |  | 3.8 | 4.9 | 3.2 | 6.9 |  | 37.2 |  |  |  |  |  | 7.0 | 9.6 |  | 5.6 |  |  | 15.1 |  |  | 2.6 |  |  | 6.1 | 13.1 | 10.1 |  | 8.4 |  | 148.2 |
| *Dicliptera bupleuroides* Nees. | Acanthaceae | Ch | Mic |  |  |  | 1.9 |  |  |  | 1.9 | 3.6 |  |  |  |  |  |  |  |  |  |  |  |  |  |  |  |  |  |  |  |  |  |  |  |  |  |  |  |  |  |  |  | 7.5 |
| *Digera muricata* (L.) Mart. | Amaranthaceae | Th | Mic |  |  |  |  |  |  |  |  |  | 8.4 |  | 3.2 |  | 3.5 |  | 4.4 |  | 1.8 |  |  |  |  |  |  |  |  |  |  |  |  |  |  |  |  |  |  |  |  |  |  | 21.4 |
| *Digitaria ciliaris* (Retz.) Koel.p | Poaceae | H | Mic |  |  |  |  |  |  |  |  |  |  |  |  |  |  |  |  |  |  |  |  |  |  |  |  |  |  |  |  | 4.7 | 41.1 | 5.5 | 9.8 |  | 25.5 |  | 7.3 |  | 10.0 |  |  | 104.0 |
| *Digiteria sanguinalis* (L.) Scop. | Poaceae | Th | Mic |  |  |  | 5.8 | 8.2 | 3.8 |  | 12.7 | 2.1 | 3.8 | 9.2 | 1.8 |  | 2.0 | 14.8 | 6.2 | 22.2 | 6.9 |  |  |  | 7.0 |  | 5.1 |  |  |  |  |  |  | 2.7 |  |  |  |  |  |  |  |  |  | 114.5 |
| *Echinochloa colona* (L.) Link. | Poaceae | Th | Mic |  |  | 4.7 | 3.7 | 7.2 | 3.4 | 9.8 | 10.4 | 5.5 | 5.6 | 7.8 | 5.5 |  |  | 20.8 |  | 24.4 |  | 7.1 | 4.2 |  |  |  |  |  |  |  |  |  |  |  |  |  |  |  |  |  |  |  |  | 120.2 |
| *Echinochloa crus-galli* (L.) P. Beauv. | Poaceae | Th | Mic |  | 16.8 | 6.2 | 22.3 | 21.1 |  | 3.7 |  |  |  |  |  |  |  |  |  |  |  |  |  |  |  |  |  |  |  |  |  |  |  |  |  |  |  |  |  |  |  |  |  | 70.0 |
| *Echinops echinatus* Roxb. | Asteraceae | Th | Mic |  |  |  |  |  |  |  |  |  |  |  |  |  |  |  |  |  |  |  |  |  |  |  | 4.3 |  |  |  |  |  |  |  |  |  |  |  |  |  | 2.8 |  |  | 7.1 |
| *Eclipta prostrata* L. | Asteraceae | H | N | 1.8 | 8.2 | 4.8 | 10.2 | 1.7 |  | 2.3 |  |  |  |  |  |  |  |  |  |  |  |  |  |  |  |  |  |  |  |  |  |  |  |  |  |  |  |  |  |  |  |  |  | 28.9 |
| *Eleocharis palustris* (L.) Roem & Schult. | Cyperaceae | G | L |  |  |  |  |  |  | 11.3 |  |  |  |  |  |  |  |  |  |  |  |  |  |  |  |  |  |  |  |  |  |  |  |  |  |  |  |  |  |  |  |  |  | 11.3 |
| *Eleusine indica* (L.) Gaertn. | Poaceae | Th | Mic |  |  |  | 9.2 | 8.3 |  |  |  |  |  |  |  |  |  |  |  |  |  |  |  |  |  |  |  |  |  |  |  |  |  |  |  |  |  |  |  |  |  |  |  | 17.5 |
| *Elodea canadensis* Michx. | Hydrocharitaceae | G | N | 1.9 | 2.6 |  |  |  |  |  |  |  |  |  |  |  |  |  |  |  |  |  |  |  |  |  |  |  |  |  |  |  |  |  |  |  |  |  |  |  |  |  |  | 4.5 |
| *Enneapogon schimperanus* (Hochst. ex A. Rich.) Renv. | Poaceae | H | Mic |  |  |  |  |  |  |  |  |  |  |  |  |  | 3.2 |  | 2.1 |  |  |  |  | 29.5 | 3.2 |  | 4.8 | 6.9 | 6.3 | 16.0 |  | 6.2 | 24.6 |  | 2.9 |  | 11.8 | 12.9 | 2.5 | 34.5 |  |  |  | 167.5 |
| *Epilobium hirsutum* L. | Onagraceae | Th | Mic |  |  | 5.1 | 7.4 |  |  |  |  |  |  |  |  |  |  |  |  |  |  |  |  |  |  |  |  |  |  |  |  |  |  |  |  |  |  |  |  |  |  |  |  | 12.5 |
| *Equisetum arvense* L. | Equisetaceae | H | Aph |  |  |  |  | 1.7 |  |  |  |  |  |  |  |  |  |  |  |  |  |  |  |  |  |  |  |  |  |  |  |  |  |  |  |  |  |  |  |  |  |  |  | 1.7 |
| *Eragrostis minor* Host. | Poaceae | Th | N |  |  |  |  |  |  |  |  |  |  | 11.1 |  | 18.1 | 8.9 | 3.9 | 18.0 | 2.3 | 3.3 | 34.0 | 23.1 |  | 22.0 | 6.3 | 4.8 |  | 21.8 | 7.6 |  | 31.2 |  | 22.3 | 7.0 |  |  | 5.3 |  | 10.1 | 7.4 |  |  | 268.7 |
| *Eragrostis papposa* (Roem. & Schult.) Steud. | Poaceae | Ch | Mic |  |  |  |  |  |  |  |  |  |  |  |  |  |  |  |  |  | 3.9 |  |  |  |  |  |  |  |  |  |  | 4.7 |  |  |  |  |  |  |  |  |  |  |  | 8.6 |
| *Erioscirpus comosus* (Wall.) Palla. | Cyperaceae | H | Mic |  |  |  |  |  |  |  |  |  |  |  |  |  |  |  |  |  |  |  |  |  |  |  |  |  |  |  |  |  |  |  |  |  |  |  |  |  |  |  | 6.5 | 6.5 |
| *Eulaliopsis binata* (Retz.) C. E. Hubbard | Poaceae | Ch | Mic |  |  |  |  |  |  |  |  |  |  |  |  |  |  |  |  |  |  |  |  |  |  |  |  |  |  |  | 3.9 |  |  |  |  | 6.4 |  |  | 32.5 | 14.9 | 68.2 | 35.2 | 5.5 | 166.5 |
| *Euphorbia granulata* Forssk. | Euphorbiaceae | Th | L |  |  |  |  |  |  |  |  |  |  |  |  | 19.1 | 6.9 |  |  |  | 1.8 |  |  |  | 1.8 | 6.6 | 2.8 |  |  |  |  |  |  |  |  |  |  |  |  |  |  |  |  | 39.1 |
| *Euphorbia heterophylla* L. | Euphorbiaceae | Th | Mic |  |  |  |  |  |  |  |  |  |  |  |  |  |  |  |  | 4.7 |  |  |  |  |  |  |  |  |  |  |  |  |  |  |  |  |  |  |  |  |  |  |  | 4.7 |
| *Euphorbia indica* Lam. | Euphorbiaceae | Th | N |  |  |  |  |  |  |  |  | 3.4 |  |  |  |  |  |  |  |  |  |  |  |  |  |  |  |  |  |  |  |  |  |  |  | 2.2 |  |  |  |  |  |  |  | 5.6 |
| *Euphorbia prostrata* Ait. | Euphorbiaceae | Th | L |  |  |  |  |  |  |  |  |  |  | 2.8 | 6.9 | 12.1 | 1.9 | 3.9 | 2.1 |  | 1.7 | 27.6 | 8.8 |  | 3.2 |  |  |  |  |  |  |  |  |  |  |  |  |  |  |  |  |  |  | 70.9 |
| *Euphorbia thymifolia* L. | Euphorbiaceae | Th | L |  | 2.4 |  |  |  | 5.8 |  |  |  |  |  | 3.5 |  |  |  |  |  |  |  |  |  |  |  |  |  |  |  |  |  |  |  |  |  |  |  |  |  |  |  |  | 11.8 |
| *Evolvulus alsinoides* (L.) L. | Convolvulaceae | H | N |  |  |  |  |  |  |  |  |  |  |  |  |  |  |  |  |  |  |  |  |  |  |  |  |  |  |  |  |  | 10.3 | 6.9 | 4.4 | 8.8 | 29.0 | 26.8 |  |  |  |  |  | 86.4 |
| *Fagonia indica* Burm. f. | Zygophyllaceae | Ch | L |  |  |  |  |  |  |  |  |  |  |  |  | 5.6 | 3.2 |  | 3.7 |  | 1.7 | 6.4 |  |  | 7.9 | 2.1 | 15.4 |  | 9.2 | 10.4 |  | 23.6 |  |  | 20.2 |  | 5.7 | 13.4 | 6.7 |  |  |  |  | 135.1 |
| *Farsetia jacquemontii* Hook. f. & Thoms. | Brassicaceae | Ch | N |  |  |  |  |  |  |  |  |  |  |  |  |  |  |  |  |  |  |  |  |  |  |  |  |  |  |  |  |  |  |  |  |  |  |  | 3.9 |  | 4.6 |  |  | 8.5 |
| *Fimbristylis dichotoma* (L.) Vahl. | Cyperaceae | G | Mic | 1.9 | 7.5 |  |  |  |  | 12.2 |  |  |  |  |  |  |  |  |  |  |  |  |  |  |  |  |  |  |  |  |  |  |  |  |  |  |  |  |  |  |  |  |  | 21.6 |
| *Forsskaolea tenacissima* L. | Urticaceae | Ch | Mic |  |  |  |  |  |  |  |  |  |  |  |  |  |  |  |  |  |  |  | 4.2 |  |  |  |  |  |  |  |  |  |  |  |  |  |  |  | 4.8 |  |  |  |  | 9.0 |
| *Heliotropium calcareum* Stocks. | Boraginaceae | H | N |  |  |  |  |  |  |  |  |  |  |  |  | 2.3 |  |  | 5.7 |  | 3.8 | 4.8 |  |  | 2.0 |  |  |  |  |  |  |  |  |  | 4.4 |  |  |  | 2.5 |  |  |  |  | 25.6 |
| *Heliotropium europaeum* L. | Boraginaceae | Th | Mic |  |  |  |  |  |  |  |  |  |  |  |  | 8.8 | 3.2 | 1.9 |  |  | 1.7 |  | 7.3 |  | 1.6 |  | 9.6 |  |  |  |  | 6.2 |  |  |  |  |  |  |  |  |  |  |  | 40.3 |
| *Heliotropium strigosum* Willd. | Boraginaceae | Th | N |  |  |  |  |  |  |  |  |  |  |  |  | 8.8 | 5.4 |  | 5.7 |  |  |  | 4.2 | 5.2 | 6.7 | 8.2 | 4.3 |  |  |  |  |  |  |  | 4.4 |  |  |  |  |  |  |  |  | 53.0 |
| *Hemarthria compressa* (L.f.) R. Br. | Poaceae | H | Mic |  |  | 16.6 | 6.5 |  | 11.8 | 2.3 |  |  |  |  |  |  |  |  |  |  |  |  |  |  |  |  |  |  |  |  |  |  |  |  |  |  |  |  |  |  |  |  |  | 37.1 |
| *Heteropogon contortus* (L.) Beauv. | Poaceae | H | Mic |  |  |  |  |  |  |  |  |  |  |  |  |  |  |  |  |  |  |  |  |  |  |  | 4.8 |  | 62.9 |  | 4.5 | 4.0 | 28.3 | 13.3 | 4.4 | 58.6 |  |  | 3.9 |  | 9.7 | 34.7 |  | 229.1 |
| *Hyparrhenia hirta* (L.) Stapf. | Poaceae | H | Mic |  |  |  |  |  |  |  |  |  |  |  |  |  |  |  |  |  |  |  |  |  |  |  |  |  |  |  | 3.9 |  |  |  |  |  |  |  |  |  |  |  |  | 3.9 |
| *Hyoscyamus insanus* Stocks. | Solanaceae | H | Mes |  |  |  |  |  |  |  |  |  |  |  |  |  |  |  |  |  |  |  |  |  |  |  |  |  |  |  |  |  |  |  | 4.4 |  |  |  |  |  |  |  |  | 4.4 |
| *Impereta cylindrica* (L.) Raeush. | Poaceae | H | N |  | 3.5 |  |  | 7.1 |  |  | 3.5 |  |  | 7.5 |  |  |  |  |  |  |  |  |  |  |  |  |  |  |  |  |  |  |  |  |  |  |  |  |  |  |  |  |  | 21.6 |
| *Indigofera linifolia* (Linn. f) Retz. | Papilionaceae | Th | N |  |  |  |  |  | 18.5 |  |  |  |  |  |  |  |  |  |  |  |  |  |  |  |  |  |  |  |  |  |  |  |  |  |  |  |  |  |  |  |  |  |  | 18.5 |
| *Ipomoea eriocarpa* R. Br. | Convolvulaceae | Th | Mic |  |  |  |  |  |  |  |  |  |  |  |  |  |  | 3.1 |  |  |  |  |  |  |  |  |  |  |  |  |  |  |  |  |  |  |  |  |  |  |  |  |  | 3.1 |
| *Ipomoea hederacea* Jacq. | Convolvulaceae | Th | Mes |  |  |  |  |  |  |  | 5.3 |  |  |  |  |  |  |  |  |  |  |  |  |  |  |  |  |  |  |  |  |  |  |  |  |  |  |  |  |  |  |  |  | 5.3 |
| *Ipomoea indica* (Burm. f.) Merrill. | Convolvulaceae | Th | Mic |  |  |  |  |  |  |  | 5.3 |  |  |  |  |  |  |  |  |  |  |  |  |  |  |  |  |  |  |  |  |  |  |  |  |  |  |  |  |  |  |  |  | 5.3 |
| *Ipomoea nil* (L.) Roth. | Convolvulaceae | Th | Mes |  |  | 4.6 |  |  |  |  |  |  |  |  |  |  |  |  |  |  |  |  |  |  |  |  |  |  |  |  |  |  |  |  |  |  |  |  |  |  |  |  |  | 4.6 |
| *Juncus compressus* Jacq. | Juncaceae | H | Mic |  |  |  |  |  |  | 4.1 |  |  |  |  |  |  |  |  |  |  |  |  |  |  |  |  |  |  |  |  |  |  |  |  |  |  |  |  |  |  |  |  |  | 4.1 |
| *Juncus maritimus* Lam. | Juncaceae | H | Mic |  |  |  |  |  |  | 6.7 |  |  |  |  |  |  |  |  |  |  |  |  |  |  |  |  |  |  |  |  |  |  |  |  |  |  |  |  |  |  |  |  |  | 6.7 |
| *Justicia peploides* T. Anders. | Acanthaceae | Th | N |  |  |  |  |  |  |  | 5.7 |  |  |  |  |  |  |  |  |  |  |  |  |  |  |  |  |  |  |  |  |  |  |  |  |  |  |  |  |  |  |  |  | 5.7 |
| *Kickxia elatine* (L.) Dumort. | Scrophulariaceae | H | N |  |  |  |  |  |  |  |  |  |  |  |  |  |  |  |  |  |  |  |  |  |  |  |  |  |  |  |  |  |  |  |  |  |  |  | 2.5 |  |  |  |  | 2.5 |
| *Kickxia ramosissima* (Wall.) Janch. | Scrophulariaceae | H | N |  |  |  |  |  |  |  |  |  |  |  |  |  |  |  |  |  |  |  |  |  |  |  |  |  |  |  |  | 4.0 |  |  | 5.4 |  |  |  | 11.5 | 8.9 | 25.4 |  | 6.5 | 61.6 |
| *Kochia indica* Wight. | Chenopodiaceae | Th | N |  |  |  |  |  |  |  |  | 3.6 | 4.4 |  | 3.2 |  |  | 5.4 |  |  | 8.2 |  |  |  |  |  |  |  |  |  |  |  |  |  |  |  |  |  |  |  |  |  |  | 24.9 |
| *Lactuca serriola* L. | Asteraceae | Th | Mic |  |  |  |  |  |  |  |  | 3.6 |  |  |  |  |  |  |  |  |  |  |  |  |  |  |  |  |  |  |  |  |  |  |  |  |  |  |  |  |  |  |  | 3.6 |
| *Salvia reflexa* Hornem. | Lamiaceae | Th | Mic |  |  |  | 3.9 |  |  |  |  |  |  |  |  |  |  |  |  |  |  |  |  |  |  |  |  |  |  |  |  |  |  |  |  |  |  |  |  |  |  |  |  | 3.9 |
| *Pervoskia arobetenoides* Karel. | Lamiaceae | Ch | Mic |  |  |  |  |  |  |  |  |  |  |  |  |  |  |  |  |  |  |  |  |  |  |  |  |  |  |  |  |  |  |  |  | 11.0 |  |  |  |  |  |  |  | 11.0 |
| *Launea procumbens* Roxb. | Asteraceae | H | Mic |  |  |  |  |  |  |  |  |  |  |  |  | 2.6 |  |  |  |  |  | 14.4 |  |  |  |  | 2.6 |  |  |  |  |  |  | 4.3 |  |  |  |  | 7.2 |  |  |  |  | 30.9 |
| *Launea secunda* Hook. f. | Asteraceae | H | Mic |  |  |  |  |  |  |  |  |  |  |  |  |  |  |  |  |  |  |  |  |  |  |  |  |  |  |  | 15.4 |  |  | 2.4 |  |  |  |  |  |  |  |  |  | 17.8 |
| *Lemna minor* L. | Lemnaceae | Th | L |  |  |  |  |  |  | 13.9 |  |  |  |  |  |  |  |  |  |  |  |  |  |  |  |  |  |  |  |  |  |  |  |  |  |  |  |  |  |  |  |  |  | 13.9 |
| *Leptochloa panicea* (Retz.) Ohwi. | Poaceae | Th | Mic |  |  |  |  |  |  |  | 11.0 |  | 1.8 |  |  |  |  |  |  |  |  |  |  |  |  |  |  |  |  |  |  |  |  |  |  |  |  |  |  |  |  |  |  | 12.8 |
| *Lespedeza juncea* (Linn. f.) Pers. | Papilionaceae | Ch | N |  |  |  |  |  |  |  |  |  |  |  |  |  |  |  |  |  |  |  |  |  |  |  |  |  |  |  | 19.9 |  |  |  |  | 8.4 |  |  |  |  |  |  |  | 28.3 |
| *Lolium perenne* L. | Poaceae | H | N |  |  |  |  |  |  |  |  |  |  |  |  |  |  |  |  |  |  |  |  |  |  | 30.3 | 2.8 |  | 9.9 |  |  |  |  |  |  |  |  |  |  |  |  |  |  | 43.1 |
| *Lycopus europaeus* L. | Lamiaceae | H | Mic | 3.9 |  | 26.2 | 9.7 |  |  |  |  |  |  |  |  |  |  |  |  |  |  |  |  |  |  |  |  |  |  |  |  |  |  |  |  |  |  |  |  |  |  |  |  | 39.8 |
| *Melhania ovata* (Cay.) Spreng. | Sterculiaceae | Th | Mic |  |  |  |  |  |  |  |  |  |  |  |  |  |  |  |  |  |  |  |  |  |  |  |  |  |  |  |  |  | 5.0 |  |  |  |  |  |  |  |  |  |  | 5.0 |
| *Malvastrum coromandelianum* (L.) Garcke. | Malvaceae | Ch | Mic |  |  | 2.0 | 11.1 | 9.6 | 7.2 |  | 33.4 | 17.3 | 6.2 |  | 4.1 |  |  | 7.3 |  | 9.2 |  |  |  |  |  |  |  |  |  |  |  |  |  | 13.9 |  |  |  |  |  |  |  |  |  | 121.2 |
| *Marsilea quadrifolia* L. | Marsileaceae | G | Mic | 12.3 | 5.9 | 1.8 | 8.7 |  |  |  |  |  |  |  |  |  |  |  |  |  |  |  |  |  |  |  |  |  |  |  |  |  |  |  |  |  |  |  |  |  |  |  |  | 28.8 |
| *Mentha longifolia* (L.) Huds. | Lamiaceae | H | Mic | 5.4 | 2.1 | 23.5 | 11.3 | 15.4 |  |  |  |  |  |  |  |  |  |  |  |  |  |  |  |  |  |  |  |  |  |  |  |  |  |  |  |  |  |  |  |  |  |  |  | 57.7 |
| *Mentha* X *piperita* L. | Lamiaceae | H | Mic |  |  |  |  |  |  | 3.7 |  |  |  |  |  |  |  |  |  |  |  |  |  |  |  |  |  |  |  |  |  |  |  |  |  |  |  |  |  |  |  |  |  | 3.7 |
| *Mollugo nudicaulis* Lam. | Molluginaceae | Th | N |  |  |  |  |  |  |  |  |  |  |  |  | 10.3 |  |  |  |  |  |  | 4.7 | 10.4 |  |  |  |  |  |  |  |  |  |  |  |  |  |  |  |  |  |  |  | 25.4 |
| *Nepeta griffithii* Hedge. | Lamiaceae | Ch | N |  |  |  |  |  |  |  |  |  |  |  |  |  |  |  |  |  |  |  |  |  |  |  |  |  |  |  | 33.5 |  |  | 4.3 | 15.5 |  |  |  |  |  |  |  |  | 53.2 |
| *Nepeta* *amicorum* Rech. F. | Lamiaceae | H | N |  |  |  |  |  |  |  |  |  |  |  |  |  |  |  |  |  |  |  |  |  |  |  |  |  |  |  |  |  |  |  |  |  |  |  |  |  |  |  | 5.5 | 5.5 |
| *Ochthochloa compressa* (Forssk.) Hilu. | Poaceae | Ch | N |  |  |  |  |  |  |  |  |  |  |  |  |  | 4.1 |  |  |  | 3.3 |  |  | 9.2 |  |  | 16.4 |  |  | 59.7 |  | 4.0 |  |  |  |  |  |  |  |  |  |  |  | 96.7 |
| *Onosma hispida* Wall. ex G. Don. | Boraginaceae | H | Mic |  |  |  |  |  |  |  |  |  |  |  |  |  |  |  |  |  |  |  |  |  |  |  |  |  |  |  | 3.9 |  |  |  |  |  |  |  |  |  |  |  |  | 3.9 |
| *Oxalis corniculata* L. | Oxalidaceae | H | N |  |  |  | 3.7 | 6.5 |  |  | 3.7 |  |  |  | 4.9 |  |  | 10.6 |  | 10.8 |  | 2.6 | 4.7 |  | 3.2 | 8.3 |  |  |  |  |  |  |  | 4.3 |  |  |  |  |  |  |  |  |  | 63.1 |
| *Panicum antidotale* Retz. | Poaceae | Ch | Mic |  |  |  |  |  |  |  |  |  | 3.8 |  |  |  |  | 7.1 |  |  | 16.1 |  |  |  | 5.6 |  |  |  |  |  |  |  |  |  |  |  |  |  |  |  |  |  |  | 32.7 |
| *Parthenium hysterophorus* L. | Asteraceae | Th | Mic | 6.6 | 7.7 |  | 6.2 | 18.5 | 13.4 |  | 20.2 | 37.8 | 26.6 | 28.1 | 14.1 | 4.9 | 7.4 | 36.6 |  | 41.0 | 18.0 |  |  |  |  | 3.9 |  |  |  |  |  |  |  | 28.4 |  |  |  |  |  |  |  |  |  | 319.6 |
| *Paspalum paspalodes* (Michx.)Scribner. | Poaceae | H | N | 40.7 | 53.5 | 32.1 | 14.1 | 22.3 |  | 31.1 |  |  |  |  |  |  |  |  |  |  |  |  |  |  |  |  |  |  |  |  |  |  |  |  |  |  |  |  |  |  |  |  |  | 193.7 |
| *Peganum harmala* L. | Zygophyllaceae | Ch | Mic |  |  |  |  |  |  |  |  |  |  |  | 12.9 |  | 3.2 |  | 8.5 |  | 10.1 | 10.0 |  |  |  |  |  |  |  |  |  |  |  |  |  |  |  |  |  |  |  |  |  | 44.7 |
| *Pennisetum orientale* L. C. Rich. | Poaceae | Ch | Mic |  |  |  |  |  |  |  |  |  |  |  |  |  |  |  |  |  |  |  | 93.6 |  |  |  |  |  |  |  |  | 11.4 | 25.6 | 2.4 | 4.1 |  | 22.9 |  | 38.3 | 34.7 | 10.9 | 73.0 | 58.3 | 375.1 |
| *Persicaria glabra* (Willd.) M. Gomes. | Polygonaceae | H | Mic | 3.6 |  | 7.9 | 11.6 | 11.5 |  |  |  |  |  |  |  |  |  |  |  |  |  |  |  |  |  |  |  |  |  |  |  |  |  |  |  |  |  |  |  |  |  |  |  | 34.6 |
| *Persicaria hydropiper* (L.) Delabre. | Polygonaceae | H | Mic | 2.2 | 12.3 | 8.3 | 26.2 | 16.5 |  |  |  |  |  |  |  |  |  |  |  |  |  |  |  |  |  |  |  |  |  |  |  |  |  |  |  |  |  |  |  |  |  |  |  | 65.6 |
| *Persicaria maculosa* Gray. | Polygonaceae | H | Mic | 15.3 |  | 2.9 |  |  |  |  |  |  |  |  |  |  |  |  |  |  |  |  |  |  |  |  |  |  |  |  |  |  |  |  |  |  |  |  |  |  |  |  |  | 18.2 |
| *Phragmites karka* (Retz.) Trin. ex Steud. | Poaceae | Ch | Mes | 20.2 | 4.0 | 9.6 |  |  |  | 7.3 |  |  |  |  |  |  |  |  |  |  |  |  |  |  |  |  |  |  |  |  |  |  |  |  |  |  |  |  |  |  |  |  |  | 41.2 |
| *Phyla nodiflora* (L.) Greene. | Verbenaceae | H | N |  | 3.9 |  |  |  | 27.6 |  |  |  |  |  |  |  |  |  |  |  |  |  |  |  |  |  |  |  |  |  |  |  |  |  |  |  |  |  |  |  |  |  |  | 31.5 |
| *Physalis divaricata* D. Don. | Solanaceae | Th | Mic |  |  |  |  |  |  |  |  |  | 14.3 |  | 3.7 |  |  |  |  |  |  |  |  |  |  |  |  |  |  |  |  |  |  |  |  |  |  |  |  |  |  |  |  | 18.0 |
| *Indigofera sp*. | Papilionaceae | H | N |  |  |  |  |  |  |  |  |  |  |  |  |  |  |  |  |  |  |  |  |  |  |  |  |  |  |  |  |  | 16.7 |  |  |  |  |  |  |  |  |  |  | 16.7 |
| *Polygala abyssinica* R.r. ex.Fresen. | Polygalaceae | Th | N |  |  |  |  |  |  |  |  |  |  |  |  |  |  |  |  |  |  |  |  |  |  |  |  |  |  |  | 4.5 |  |  |  |  |  |  |  |  |  |  |  |  | 4.5 |
| *Polygala arvensis* Willd. | Polygalaceae | Th | N |  |  |  |  |  |  |  |  |  |  |  |  |  |  |  |  |  |  |  |  | 9.6 | 6.9 | 6.5 |  |  |  |  |  |  |  |  |  | 13.6 | 11.8 | 31.1 |  |  |  |  |  | 79.5 |
| *Polygala erioptera* DC. | Polygalaceae | Th | N |  |  |  |  |  |  |  |  |  |  |  |  |  |  |  |  |  |  |  |  |  |  |  |  |  |  |  |  |  |  |  | 2.3 | 4.5 |  |  |  |  |  |  |  | 6.7 |
| *Polygonum aviculare* L. | Polygonaceae | Th | N |  |  |  | 3.7 |  | 6.6 |  |  | 11.0 |  |  |  |  |  |  |  |  |  |  |  |  |  |  |  |  |  |  |  |  |  |  |  |  |  |  |  |  |  |  |  | 21.3 |
| *Polygonum plebjum* R. Br. | Polygonaceae | H | L |  |  |  |  |  | 8.9 |  |  | 2.1 |  |  | 6.5 |  |  | 1.8 |  |  |  |  |  |  |  |  |  |  |  |  |  |  |  |  |  |  |  |  |  |  |  |  |  | 19.2 |
| *Portulaca oleracea* L. | Portulacaceae | Th | N |  |  |  |  |  |  |  |  | 3.6 | 1.6 |  | 2.9 |  | 2.0 |  |  |  | 1.7 |  |  |  |  |  |  |  |  |  |  |  |  |  |  |  |  |  |  |  |  |  |  | 11.9 |
| *Portulaca pilosa* L. | Portulacaceae | H | L |  |  |  |  |  |  |  |  |  |  |  |  |  |  |  |  |  | 3.6 |  |  |  |  |  |  | 12.9 |  |  |  |  |  |  |  |  |  |  |  |  |  |  |  | 16.5 |
| *Potamogeton nodosus* Poiret. | Potamogetonaceae | H | Mic | 1.9 |  |  |  |  |  | 4.2 |  |  |  |  |  |  |  |  |  |  |  |  |  |  |  |  |  |  |  |  |  |  |  |  |  |  |  |  |  |  |  |  |  | 6.1 |
| *Potentilla supina* L. | Rosaceae | H | N |  |  |  |  |  | 2.3 |  |  |  |  |  |  |  |  |  |  |  |  |  |  |  |  |  |  |  |  |  |  |  |  |  |  |  |  |  |  |  |  |  |  | 2.3 |
| *Sesuvium sesuvioides* (Fenzl) Verdc. | Aizoaceae | Th | N |  |  |  |  |  |  |  |  |  |  |  |  |  |  |  | 8.1 |  |  |  |  |  |  |  |  |  |  |  |  |  |  |  |  |  |  |  |  |  |  |  |  | 8.1 |
| *Pseudgaillonia hymenostephana* (Daub. & Spach) Lincz. | Rubiaceae | Ch | N |  |  |  |  |  |  |  |  |  |  |  |  |  |  |  |  |  |  |  |  |  |  |  |  |  |  |  |  |  |  |  | 18.4 |  | 23.2 | 10.5 |  |  |  |  |  | 52.1 |
| *Pulicaria undulata* (L.) Mey. | Asteraceae | Th | Mic |  |  |  |  |  | 33.2 |  |  |  |  |  |  |  |  |  |  |  |  |  |  |  |  |  |  |  |  |  |  |  |  |  |  |  |  |  |  |  |  |  |  | 33.2 |
| *Pupalia lappaceae* (L.) Juss. | Amaranthaceae | Ch | Mic |  |  |  |  |  |  |  |  |  |  |  |  |  |  |  | 14.3 |  |  |  |  |  |  |  |  |  |  |  |  |  | 16.9 |  |  |  | 8.6 | 11.3 |  |  |  |  | 14.9 | 66.0 |
| *Pycreus flavidus* T. Koyama. | Cyperaceae | G | Mic |  | 8.2 |  |  |  |  | 14.6 |  |  |  |  |  |  |  |  |  |  |  |  |  |  |  |  |  |  |  |  |  |  |  |  |  |  |  |  |  |  |  |  |  | 22.8 |
| *Rhynchosia capitata* (Heyne ex Roth.) DC. | Papilionaceae | Th | Mic |  |  |  |  |  |  |  |  |  |  |  |  |  | 12.9 |  |  |  |  |  |  |  |  |  |  |  |  |  |  |  |  |  |  |  |  |  |  |  |  |  |  | 12.9 |
| *Rhynchosia minima* (L.) DC. | Papilionaceae | H | Mic |  |  |  |  |  |  |  |  |  |  |  |  |  | 6.4 |  | 9.9 |  | 3.0 |  |  |  | 9.8 | 4.2 | 9.0 |  |  |  |  |  | 16.7 | 5.3 | 4.4 |  |  | 5.3 |  |  |  |  | 6.5 | 80.4 |
| *Ruellia tuberosa* L. | Acanthaceae | Th | Mic |  |  |  |  | 7.5 |  |  | 26.6 | 6.7 | 3.8 |  |  |  |  |  |  |  |  |  |  |  |  |  |  |  |  |  |  |  |  |  |  |  |  |  |  |  |  |  |  | 44.6 |
| *Rumex hastatus* D. Don. | Polygonaceae | Ch | Mic |  |  |  |  |  |  |  |  |  |  |  |  |  |  |  |  |  |  |  |  |  |  |  |  |  |  |  |  |  |  | 10.8 |  |  |  |  |  |  |  |  |  | 10.8 |
| *Saccharum filifolium* Steud. | Poaceae | H | Mic |  |  |  |  |  |  |  |  |  |  | 7.5 |  |  |  |  |  |  |  |  |  |  |  |  |  |  |  |  |  |  |  |  |  |  |  |  |  |  |  |  |  | 7.5 |
| *Saccharum spontaneum* L. | Poaceae | H | Mic | 14.1 | 30.1 |  |  |  | 3.2 | 19.5 |  |  |  | 35.9 |  |  |  |  |  | 11.9 |  |  |  |  |  |  |  |  |  |  |  |  |  |  |  |  |  |  |  |  |  |  |  | 114.7 |
| *Sagittaria trifolia* L. | Alismataceae | G | Mes | 7.8 |  |  |  |  |  |  |  |  |  |  |  |  |  |  |  |  |  |  |  |  |  |  |  |  |  |  |  |  |  |  |  |  |  |  |  |  |  |  |  | 7.8 |
| *Salsola tragus* L. | Chenopodiaceae | Th | N |  |  |  |  |  |  |  |  |  |  |  |  |  |  |  |  |  |  |  |  |  | 3.2 |  |  |  |  |  |  |  |  |  |  |  |  |  |  |  |  |  |  | 3.2 |
| *Salvia aegyptiaca* L. | Lamiaceae | Ch | N |  |  |  |  |  |  |  |  |  |  |  |  |  |  |  |  |  |  |  |  | 9.6 |  | 14.6 | 6.8 |  |  | 4.2 |  | 4.5 |  |  | 2.6 |  |  |  |  |  |  |  |  | 42.3 |
| *Salvia moocroftiana* Wall. ex Benth. | Lamiaceae | H | Mes |  |  |  |  |  |  |  |  |  |  |  |  |  |  |  |  |  | 3.6 |  |  |  |  |  |  |  |  |  |  |  |  |  |  |  |  |  |  |  |  |  |  | 3.6 |
| *Scabiosa candollei* DC. | Dipsacaceae | Th | N |  |  |  |  |  |  |  |  |  |  |  |  |  |  |  |  |  |  |  |  |  |  |  |  |  |  |  | 2.3 |  |  |  |  |  |  |  |  |  |  |  |  | 2.3 |
| *Scrozonera virgata* DC. | Asteraceae | Ch | Mic |  |  |  |  |  |  |  |  |  |  |  |  |  |  |  |  |  |  |  |  |  |  |  |  |  |  |  |  |  |  |  | 4.4 |  |  |  |  |  | 4.3 |  |  | 8.8 |
| *Scutellaria linearis* Benth | Lamiaceae | H | N |  |  |  |  |  |  |  |  |  |  |  |  |  |  |  |  |  |  |  |  |  |  |  |  |  |  |  | 16.3 |  |  | 4.3 |  |  |  |  |  |  |  |  |  | 20.6 |
| *Senna occidentalis* (L.) Link. | Caesalpinaceae | Th | Mic |  |  |  |  |  |  |  |  |  | 4.9 |  |  |  |  |  |  |  |  |  |  |  |  |  |  |  |  |  |  |  |  |  |  |  |  |  |  |  |  |  |  | 4.9 |
| *Senn*a *sp* | Caesalpiniaceae | Ch | Mic |  |  |  |  |  |  |  |  |  |  |  |  |  |  |  |  |  |  |  |  |  | 3.2 |  |  |  |  |  |  |  |  |  |  |  |  |  |  |  |  |  |  | 3.2 |
| *Sesbania sesban* (L.) Merrill | Papilionaceae | Th | N |  |  |  |  |  | 5.5 |  |  |  | 3.2 |  |  |  |  |  |  |  |  |  |  |  |  |  |  |  |  |  |  |  |  |  |  |  |  |  |  |  |  |  |  | 8.7 |
| *Setaria pumila* (Poir.) Roem. & Schult. | Poaceae | Th | Mic | 4.8 | 2.1 | 7.0 | 9.1 | 14.4 |  | 2.3 | 5.1 | 7.0 |  |  |  |  |  |  |  | 4.6 |  |  |  |  |  |  |  |  |  |  |  |  |  | 20.9 |  | 4.4 |  |  |  |  |  |  |  | 81.8 |
| *Setaria* *verticillata* P. Beauv. | Poaceae | Th | Mic |  |  |  |  |  |  |  | 15.9 | 7.5 | 3.2 |  |  |  |  |  |  |  |  |  |  |  |  |  |  |  |  |  |  |  |  |  |  |  |  |  |  |  |  |  |  | 26.6 |
| *Setaria viridis* (L.) P. Beauv. | Poaceae | Th | Mic |  |  |  |  |  |  |  |  |  |  |  |  |  |  |  |  |  |  |  |  |  |  |  |  |  |  |  |  |  |  | 17.6 |  |  |  |  |  |  |  |  |  | 17.6 |
| *Shoenoplectus litoralis* (Schard.) Palla. | Cyperaceae | G | Mes | 24.6 | 4.2 |  |  |  |  | 4.3 |  |  |  |  |  |  |  |  |  |  |  |  |  |  |  |  |  |  |  |  |  |  |  |  |  |  |  |  |  |  |  |  |  | 33.2 |
| *Sida cordifolia* L. | Malvaceae | Ch | Mic |  |  |  |  |  |  |  |  |  |  |  |  |  |  |  |  |  |  |  |  |  |  |  |  |  |  |  |  |  |  | 4.3 |  |  |  |  |  |  |  |  |  | 4.3 |
| *Sida ovata* Forssk. | Malvaceae | Ch | Mic |  |  |  |  |  | 4.7 |  |  |  |  |  |  |  |  |  |  |  |  |  |  |  |  |  |  |  |  |  |  |  | 4.6 |  |  |  |  |  |  |  |  |  |  | 9.3 |
| *Solanum nigrum* L. | Solanaceae | Ch | Mic | 3.3 |  | 6.1 | 3.7 |  |  |  |  |  |  |  | 3.2 |  | 1.9 |  |  |  | 1.8 | 2.6 |  |  |  |  |  |  |  |  |  |  |  |  |  |  |  |  |  |  | 4.6 |  |  | 27.1 |
| *Solanum surattense* Burm. f. k. | Solanaceae | Th | Mic |  |  |  |  |  | 7.0 |  |  |  |  | 4.8 | 8.6 | 20.3 | 3.0 | 2.9 | 9.7 |  | 1.8 | 15.6 | 4.2 |  | 6.3 | 2.3 | 8.5 |  |  |  |  | 2.0 |  |  | 15.0 |  |  |  | 6.5 |  | 21.9 |  |  | 140.5 |
| *Sonchus wighthianus* DC. | Asteraceae | Ch | Mic |  |  | 1.8 | 1.9 | 1.6 |  |  |  |  |  |  |  |  |  |  |  |  |  |  |  |  |  |  |  |  |  |  |  |  |  |  |  |  |  |  |  |  |  |  |  | 5.3 |
| *Sorghum halepense* (L.) Pers. | Poaceae | H | Mic |  |  |  |  |  |  |  | 6.1 | 3.6 | 9.5 |  | 6.8 |  | 7.1 |  |  |  | 3.8 |  |  |  |  |  |  |  |  |  |  |  |  |  |  |  |  |  |  |  |  |  |  | 36.8 |
| *Stachys parviflora* Benth. | Lamiaceae | Ch | Mic |  |  |  |  |  |  |  |  |  |  |  |  |  |  |  |  |  |  |  |  |  |  | 18.3 |  |  |  |  |  |  |  |  |  |  |  |  |  |  |  |  |  | 18.3 |
| *Tagetes minuta* L. | Asteraceae | Th | Mic |  |  |  |  |  |  |  |  |  |  |  |  |  |  |  |  |  |  |  |  |  |  |  |  |  |  |  |  |  |  | 9.0 |  |  |  |  |  |  |  |  |  | 9.0 |
| *Tetrapogon villosus* Desf. | Poaceae | Ch | Mic |  |  |  |  |  |  |  |  |  |  |  |  |  |  |  | 7.1 |  |  |  |  |  |  | 7.7 | 9.0 |  | 5.6 | 12.5 |  | 29.8 | 12.9 | 4.8 | 23.3 |  | 12.9 |  | 28.9 | 24.0 | 28.4 | 4.5 | 27.3 | 238.8 |
| *Teucrium stocksianum* Boiss. | Lamiaceae | Ch | N |  |  |  |  |  |  |  |  |  |  |  |  |  |  |  |  |  |  |  |  |  |  | 17.0 |  |  |  |  | 12.3 |  |  |  |  |  |  |  |  | 42.0 |  |  |  | 71.3 |
| *Themeda anathera* (Nees ex Steud.) Hack. | Poaceae | Ch | Mic |  |  |  |  |  |  |  |  |  |  |  |  |  |  |  |  |  |  |  |  |  |  |  |  |  |  |  | 47.9 |  |  | 6.3 |  |  |  |  |  |  |  |  |  | 54.3 |
| *Tragus roxburghii* Panigrahi. | Poaceae | Th | N |  |  |  |  |  |  |  |  | 2.1 |  |  |  | 6.7 | 7.1 | 3.4 | 16.4 | 3.9 | 4.0 | 5.7 |  | 5.6 | 13.3 | 8.5 | 8.5 |  |  |  |  | 2.5 |  |  |  |  |  |  |  |  |  |  |  | 87.7 |
| *Trianthema portulacastrum* L. | Aizoaceae | Th | Mic |  |  |  |  |  |  |  | 5.1 | 13.0 | 9.8 |  | 3.8 |  |  |  |  |  |  |  |  |  |  |  |  |  |  |  |  |  |  |  |  |  |  |  |  |  |  |  |  | 31.7 |
| *Tribulus terrestris* L. | Zygophyllaceae | Th | N |  |  |  |  |  |  |  |  | 5.8 | 8.5 |  | 7.9 | 8.8 | 9.7 | 6.8 | 13.1 |  | 5.1 | 4.8 |  |  | 6.8 | 8.9 | 4.8 |  |  |  |  |  |  |  |  |  |  |  |  |  |  |  |  | 90.9 |
| *Trichodesma indicum* (L.) R. Br. | Boraginaceae | H | N |  |  |  |  |  |  |  |  |  |  |  |  |  |  |  |  |  |  |  |  |  |  | 4.3 |  |  |  |  |  |  |  |  |  |  |  |  |  |  |  |  |  | 4.3 |
| *Tricholaena teneriffae* (L.F.) Link. | Poaceae | H | Mic |  |  |  |  |  |  |  |  |  |  |  |  |  |  |  |  |  |  |  |  |  |  |  |  |  |  |  |  |  |  |  | 5.4 |  | 7.9 |  |  |  |  |  |  | 13.3 |
| *Typha domingensis* Pers. | Typhaceae | G | Meg | 46.5 | 14.8 |  |  |  |  | 38.0 |  |  |  |  |  |  |  |  |  |  |  |  |  |  |  |  |  |  |  |  |  |  |  |  |  |  |  |  |  |  |  |  |  | 99.2 |
| *Typha latifolia* L. | Typhaceae | G | Meg |  | 4.7 |  |  |  |  | 7.6 |  |  |  |  |  |  |  |  |  |  |  |  |  |  |  |  |  |  |  |  |  |  |  |  |  |  |  |  |  |  |  |  |  | 12.3 |
| *Vallisneria spiralis* L. | Hydrocharitaceae | G | N | 5.1 |  |  |  | 3.1 |  |  |  |  |  |  |  |  |  |  |  |  |  |  |  |  |  |  |  |  |  |  |  |  |  |  |  |  |  |  |  |  |  |  |  | 8.2 |
| *Verbena officinalis* L. | Verbenaceae | H | Mic |  |  |  |  | 3.2 |  |  |  | 5.8 | 4.7 |  | 9.1 | 4.2 | 3.5 |  |  |  |  | 10.9 |  |  |  |  |  |  |  |  |  |  |  |  |  |  |  |  |  |  |  |  |  | 41.4 |
| *Verbesina encelioides* (Cav.) Benth & Hook. F. ex A. Gray. | Asteraceae | Th | Mic |  |  |  |  |  |  |  |  |  |  | 13.9 |  |  |  | 5.1 |  |  | 6.9 |  |  |  | 6.9 |  |  |  |  |  |  |  |  |  |  |  |  |  |  |  |  |  |  | 32.9 |
| Unknown |  | Ch | N |  |  |  |  |  |  |  |  |  |  |  |  |  |  |  |  |  |  |  |  |  |  |  |  |  |  |  |  |  | 9.4 |  |  |  |  |  |  |  |  |  |  | 9.4 |
| *Xanthium strumarium* L. | Asteraceae | Th | Mes | 23.3 | 15.0 |  | 6.3 | 1.7 | 15.0 |  | 4.0 | 5.8 | 4.5 | 4.8 | 3.2 | 5.4 | 14.2 | 3.1 |  |  |  |  |  |  |  |  |  |  |  |  |  |  |  |  |  |  |  |  |  |  | 5.4 |  |  | 111.7 |
| *Zaleya pentandra* (L.) Jeffrey. | Aizoaceae | Th | Mic |  |  |  |  |  |  |  |  |  |  |  | 3.2 |  | 3.5 | 3.7 | 7.8 |  | 5.8 |  |  |  |  |  |  |  |  |  |  |  |  |  |  |  |  |  |  |  |  |  |  | 24.1 |
